# Sexual and reproductive health outcomes are positively associated with comprehensive sexual education exposure in Mexican high-school students

**DOI:** 10.1371/journal.pone.0193780

**Published:** 2018-03-19

**Authors:** Filipa de Castro, Rosalba Rojas-Martínez, Aremis Villalobos-Hernández, Betania Allen-Leigh, Ariela Breverman-Bronstein, Deborah Lynn Billings, Patricia Uribe-Zúñiga

**Affiliations:** 1 Center for Population Health Research, National Institute of Public Health, Cuernavaca, Mexico; 2 Choose Well Initiative. Columbia, South Carolina, United States of America; 3 National Center for HIV and AIDS Control and Prevention, Mexico City, Mexico; University of Westminster, UNITED KINGDOM

## Abstract

**Objectives:**

To describe the comprehensive sex education (CSE) coverage and to evaluate a set of indicators related to knowledge, attitudes, and practices associated with the seven components of the CSE framework among Mexican high-school students.

**Methods:**

We conducted a cross-sectional survey of a nationally representative sample of students in public and private high schools in Mexico. Questions about CSE coverage and about knowledge, attitudes, and practices related to sexual health were included. We present descriptive statistics for demographic characteristics, exposure to CSE, and sexual health outcomes. We fitted a series of multivariate logistic regression models to examine the association between each CSE component exposure and sexual health outcomes, adjusting for age and sex.

**Results:**

There were significant associations between being exposed to each CSE components and the related sexual health outcomes. The strongest one was for identifying effective contraceptives among those exposed to the Sexual and Reproductive Health component (SRH) (OR 4.10; 95%CI[2.93,5.75]). Also, students exposed to the relationships component had 20% higher odds of affirming they could convince their partner to use condoms (OR 1.20; 95%CI[1.05,1.36]).

**Conclusions:**

This paper provides evidence of the potential beneficial effects of CSE on attitudes, knowledge, and behaviors regarding sexual and reproductive health among adolescents. In addition, it identifies areas that should be strengthened to increase the positive impact of CSE.

## Introduction

Worldwide, adolescent sexual and reproductive health remains a challenge. The new Sustainable Development Goals agenda reflects the importance of youth citizenship, autonomy, rights and active and healthy engagement in society as core youth development aspects. Yet, despite governmental efforts, the health and wellbeing needs of many adolescents are not being fulfilled [1]. Sixteen million births worldwide, 111 million cases of sexually transmitted infections (STI), and 15% of new adult HIV cases, occur among adolescents [2–4]. Worldwide, maternal health issues are a leading cause of death among adolescent women [5]. In 2015 the Adolescent Fertility Rate (AFR) for 15 to 19 years old women was 44 per 1000 births, with much higher rates in low-income counties (97 per 1000) compared to high-income countries (19 per 1000) [5]. Mexico, a middle-income country (LMIC), has systematically occupied the first place for AFR among Organization for Economic Cooperation and Development (OECD) members, with a birth rate of 64.2 per 1000 births in 15–19 years-old women in 2008, which by 2014 had increased to 77 per 1000 births [6,7].

Improving sexual and reproductive health outcomes in adolescents requires interventions that support them during their first sexual encounters, addressing both individual and social determinants that might influence their reproductive health choices and experiences [8]. Comprehensive Sexual Education (CSE), as defined by the International Planned Parenthood Federation (IPPF) seeks to provide adolescents the opportunity to explore their knowledge and attitudes towards sex and sexuality, and empower them to make informed decisions about their sex life [2,9]. CSE, under the IPPF framework, involves 7 basic components: Gender, Sexual and Reproductive Health and HIV (SRH), Sexual Rights and Sexual Citizenship, Pleasure, Violence, Diversity, and Relationships [2]. A recent meta-analysis found that CSE decreases the risk associated with HIV, increasing HIV knowledge and self-efficacy associated to condom use, and frequency of condom use. Furthermore, students who receive CSE report having better knowledge and feeling more prepared to face important decisions regarding their health [10].

In Mexico, as in other LMIC despite the efforts to consolidate a CSE curriculum, there is little evidence about its coverage, comprehensiveness and impact. This paper aims to describe the CSE coverage and to evaluate its association with a set of indicators related to sexual and reproductive health knowledge, attitudes, and practices based on the seven CSE components, in a representative sample of Mexican high-school students.

## Methods

### Survey developed

To gather information about the seven CSE components defined by IPPF, we designed a cross-sectional survey and applied it to a probabilistic, nationally representative sample of students enrolled in public and private high-schools in Mexico, stratified by clusters. The response rate was 66%. Schools not agreeing to participate were replaced using a matching procedure for the selection of substitute schools; greater detail about the sampling design can be consulted in Rojas 2017 [11]. In each of the 45 randomly selected schools, we then randomly selected one group from first year students, one from second year, and one from third year, regardless of the shift (morning or afternoon). Altogether 3,824 adolescents, aged 15 to 18 years who agreed to participate, were included in the study.

### Process

The study was approved by the ethics review board of the National Institute of Public Health in Mexico. To proceed with school and student recruitment we first presented the study to the staff of the selected schools. In those schools deciding to participate in the study, we then invited selected students to participate. We obtained written consent from the parents or legal guardians for students younger than 18 years, and verbal consent from students 18 years old; before answering the survey, the computer software asked the students if they agreed to complete the questionnaire in order to proceed. The survey was conducted from October to November 2014. Students, organized in 6–8 students’ groups, individually completed questionnaires in private on-site rooms, supervised by fieldwork personnel.

### Study instrument and variables

To reduce the risk of errors during application, ensuring standardization, privacy and confidentiality of interviews, the questionnaire was self-administered using a computerized audio system (Audio Computer-Assisted Self-Interview Software, ACASI). The study instrument was organized in 3 main sections. The first section captured basic socio-demographic data, such as age, sex, marital status, past and current education. On the second section we addressed exposure to CSE, by asking about information on a list of 23 topics organized according to the seven IPPF components (Table 1). The following question was used to assess CSE coverage: “on any occasion during primary, secondary or high-school, did a school teacher, school counselor or school head talk to you about (CSE topic)”. If students responded positively, it was considered that the CSE topic had been covered. If students responded positively to all CSE topics within a component, it was considered that the CSE component had been covered.

**Table 1:** Subtopics students were asked about to measure exposure to comprehensive sex education (CSE) topics in high-school students at any time during schooling, Mexico, 2014.

Component	Subtopic
*Gender*	
	Gender equity
	Relationships (gender roles)
*Sexual and Reproductive Health and HIV*	
	The reproductive system
	Puberty
	How to prevent pregnancy
	How to prevent HIV
	How to prevent STIs
	Condom use and negotiation (discussion in general)
	Correct use of condoms and other contraceptive methods
*Sexual Rights and Sexual Citizenship*	
	Reproductive and sexual rights
	Access to health services
	Where to get condoms and other contraceptive methods
	How to overcome barriers to getting condoms or other contraceptive methods
*Pleasure*	
	Pleasure related to sex
*Violence*	
	Physical violence
	Sexual violence
*Diversity*	
	Respect for diversity and disability
	Respect for people with HIV/AIDS
	Respect for sexual orientation
	Respect for ethnic origins
*Relationships*	
	How to talk to your partner about using condoms
	Values and social relations, recognition of healthy and coercive relationships
	How to avoid situations where you could be forced to have sex or to have unprotected sex.

On the third section of the instrument, we collected information about sexual and reproductive health knowledge, attitudes, and practices of students according to a set of indicators related to the 7 components of CSE (Table 2) (for more information consult S1 and S2 Appendices).

**Table 2:** Description of outcomes related to comprehensive sex education (CSE) components, Mexico, 2014.

Indicator	Description
**Gender**	
*Gender-equitable attitudes*	Adolescents who have at least two out of six of the following gender-equitable attitudes: 2 questions about whether violence in relationships between men and women is acceptable; 1 question about whether contraception is principally a woman’s responsibility; 1 question about whether men need sex more than women; and giving the same answer (yes to both or no to both) as to whether a woman or a man who has sex before marriage will regret it. Disagreeing with the first 4 questions and giving the same answer to the last two questions was scored as a higher level of gender-equitable attitudes.
**Sexual and Reproductive Health and HIV**	
*Recognize at least three out of five effective contraceptives*	To measure recognition of effective contraceptives we asked, Have you heard of (each of the following contraceptive methods was listed): contraceptive pills, contraceptive injections, condoms, IUD, contraceptive patch? We constructed a dichotomous variable classifying adolescents who recognize 3 or more of these methods and adolescents who recognize less than 3.
*Have correct knowledge on at least five out of nine of the SRH topics*	Adolescents who correctly answered 5 or more out of 9 questions about sexual and reproductive health such as the following: if a condom can be used more than once, if a condom can slip off the penis and get lost in a woman’s body, if condoms protect against HIV/AIDS, against sexually transmitted infections, against pregnancy, how many hours after having sex can emergency contraception be used (24 hours, 120 hours or a month, where 120 hours was the correct answer).
*Know that condoms can only be used once*	Adolescents who answer correctly that a condom can only be used once
*Have positives attitudes towards condoms*	Adolescents who have at least three of the following positive attitudes towards condom use: responding that condoms are a method that is appropriate for adolescents to use in occasional relationships; in stable loving relationships; that they would not be ashamed to buy condoms; that suggesting condom use does not imply a lack of trust and responding that condoms can or should be used during sex after a couple is married.
**Sexual Rights and Citizenship**	
*Know at least two out of four of their SRH rights*	Adolescents who answered that at least two of the following are sexual and reproductive health rights of adolescents: receiving condoms, receiving contraceptive methods, receiving emergency contraception, having sex only when or if they want to.
*Sought sexual and reproductive health counseling*	Adolescents who report they went to a health facility in the last year seeking any sexual or reproductive health (SRH) service or information
**Pleasure**	
Have positive attitudes towards sexual pleasure	Adolescents who identify at least four positive attitudes towards pleasure, such as agreeing that it is acceptable for adolescents who are not married to have a boyfriend or girlfriend, have dates, kiss, hug, and touch each other, among others.
Have positive attitudes towards sexuality	Adolescents who identify at least five positive attitudes towards sexuality such as agreeing that it is acceptable for adolescents to have sex if they love each other or that it is acceptable for two adolescents to have sex before marriage, to see if they are compatible, among others.
**Violence**	
*Suffered family violence over the last year*	Adolescents who report suffering any kind of violence perpetrated by a family member over the last year: if you father, mother, brother, sister or another person living in your home insulted you, called you names, made you feel fear, slapped, hit or punched you in the last year.
*Suffered dating violence over the last year*	Adolescents who report suffering any kind of partner or dating violence over the last year including: if a partner, boyfriend or girlfriend insulted you, called you stupid, made fun of you, prohibited you from having friends, spread false rumors abut you, or hurt you physically on purpose, or did something sexual to you when you didn’t want to, in the last year.
*Suffered sexual abuse (in their lifetime)*	Adolescents who report any sexual abuse (if someone has ever touched your genitals or done sexual things to you when you didn’t want to) at any time in their lives
*Suffered bullying at school over the last year*	Adolescents who report being bullying victims over the last year, measured by asking how often in the last year has someone done the following to them at school: insulted you or used foul language towards you, made you feel bad or feel fear, slapped, hit or punched you.
**Diversity**	
*During the last year*, *suffered bullying because of*:	
*The color of their skin*, *disability*, *or religion*	Adolescents who report any violence (you felt unsafe at school, you have been called ugly names, threatened, pushed or hit) in school over the last year, because of your gender, skin color, a disability or because they think you have a disability, because of your religion.
*During the last year*, *suffered bullying because of*: *The way they express their gender*	Adolescents who report violence in school (you felt unsafe at school, you have been called ugly names, threatened, pushed or hit) because of how they express their gender or how traditionally “masculine” or “feminine” they are in terms of how they look or act, over the last year.
*During the last year*, *suffered bullying because of*: *Homophobic bullying*	Adolescents who report violence (you felt unsafe at school, you have been called ugly names, threatened, pushed or hit) because of their sexual orientation over the last year.
**Relationships**	
*Make more than half of the decisions in their relationship*	In adolescents who reported ever having had a girlfriend, boyfriend or partner, equitable decision-making was measured by asking who made the following decisions: when to visit friends; participate in school activities; go to the movies, go out in general, go to parties; whether to skip a class; what type of clothes the adolescent him or herself should use. A higher score was assigned when the adolescent responded that decisions were made by both members of the couple, except for what clothes the adolescent should wear, which was given a higher score (for equitable decisions) if the adolescent decided him or herself.
*Can talk about using condoms with their partner*	Adolescents who report feeling sure or very sure they could talk to their partners about using condoms
*Can say they don’t want to have sex unless their partner uses a condom*	Adolescents who report feeling sure or very sure they could say they don’t want to have sex without a condom
*Say they could convince their partner to use condoms*	Adolescents who report feeling sure or very sure that they could convince their partner to use a condom
*Negotiated condom use in their first sexual encounter*	Adolescents who report talking to their partners about contraceptive use before or during their first sexual encounter.

### Data analysis

Sample weights for each study participant were estimated, considering the probability of selection and non-response rate. We obtained descriptive analysis and confidence intervals at 95% (95% CI) for demographic characteristics, exposure to CSE topics according to the IPPF seven component framework, and sexual and reproductive health outcomes (knowledge, attitudes, and practices). All results were adjusted for the sampling design of the survey.

**Fig 1:**
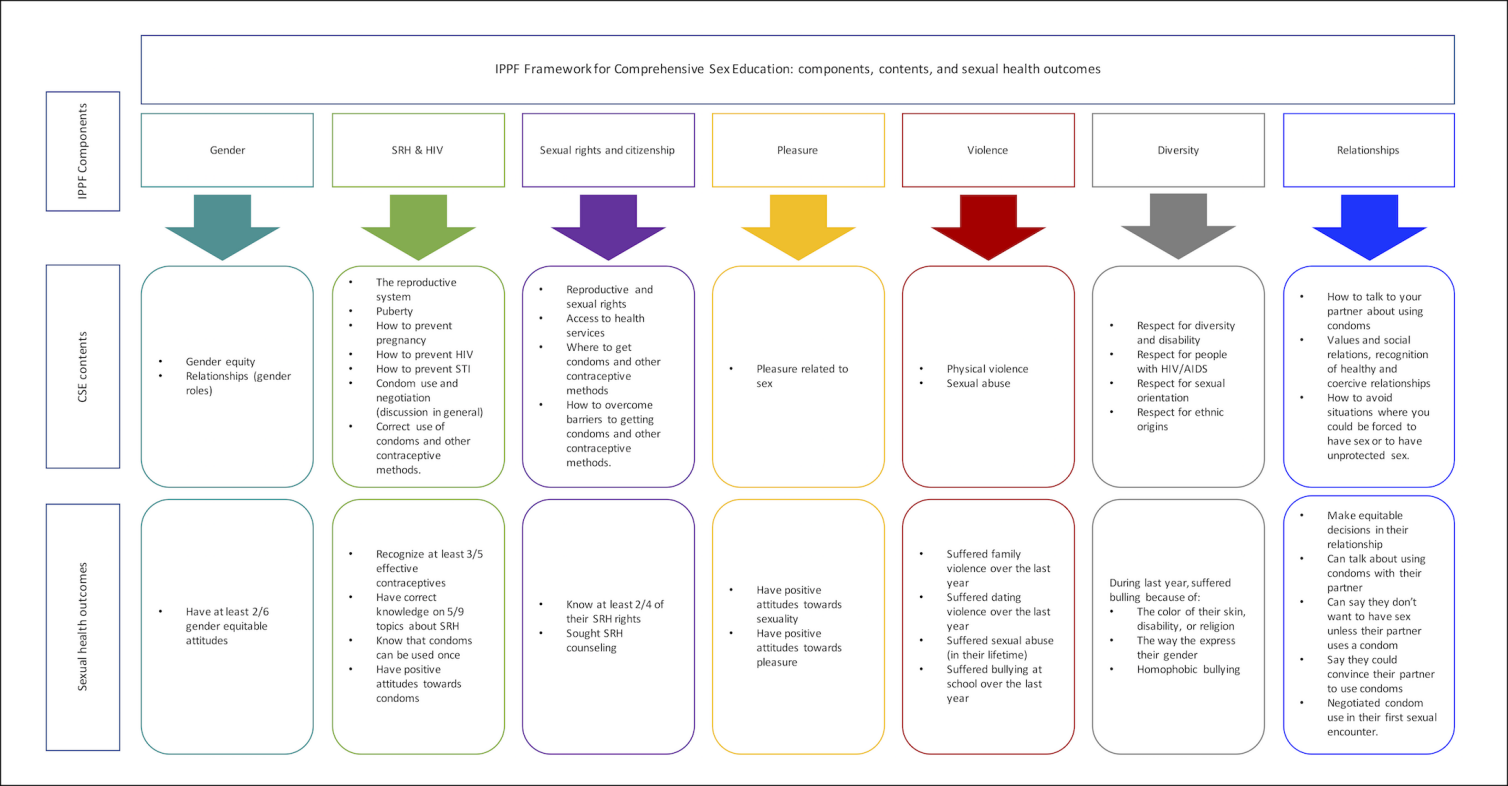
Conceptual framweork of the association between the IPPF framework, CSE components and sexual health outcomes.

To estimate the association between sexual and reproductive health outcomes with exposure to CSE components, we fitted a series of multivariate logistic regression models adjusted for age and sex. For each model the dependent variable was the sexual and reproductive health outcome while the predictor was exposure to all of the subtopics within the CSE component that the outcome corresponded to (Fig 1). All statistical analysis were performed using the SVY command, to account for the complex survey design, with the statistical package Stata 13.1 (College Station, TX: StataCorp LP).

## Results

Population characteristics are summarized in Table 3. Approximately half of the students (54.6%) were 15 to 16 years-old, most of them were single (96.7%) and attended public schools (81.1%), with no differences between males and females.

**Table 3:** Population characteristics by gender. Mexico 2014.

	Male % [95%CI]	Female % [95%CI]	Total % [95%CI]
**Age**			
15 to 16	55.3 [50.4,60.0]	54.0 [49.1,58.8]	54.6 [50.3,58.8]
17 to 18	44.7 [40.0,49.6]	46.0 [41.2,50.9]	45.4 [41.2,49.7]
**Marital Status**			
Single	96.1 [95.0,96.9]	97.3 [96.5,97.9]	96.7 [96.1,97.2]
Married/cohabitation	3.6 [2.8,4.7]	2.4 [1.8,3.1]	3.0 [2.5,3.6]
Separated/divorced/widowed	0.3 [0.1,0.7]	0.3 [0.1,0.8]	0.3 [0.2,0.6]
**School grade**^A^			
First year	36.1 [31.9,40.5]	30.4 [25.3,36.0]	33.2 [29.0,37.6]
Second year	33.5 [30.0,37.1]	33.9 [30.0,38.0]	33.7 [30.6,36.9]
Third year	30.4 [26.9,34.1]	35.7 [30.4,41.3]	33.2 [29.4,37.1]
**Type of school**			
Public	83.6 [80.3,86.4]	78.9 [74.6,82.6]	81.1 [77.8,84.1]
Private	16.4 [13.6,19.7]	21.1 [17.4,25.4]	18.9 [15.9,22.2]
**Type of area where school is located**			
Urban	98.3 [87.8,99.8]	98.2 [87.4,99.8]	98.3 [87.6,99.8]
Rural	1.7 [0.2,12.2]	1.8 [0.2,12.6]	1.7 [0.2,12.4]

Proportion of students reporting exposure to CSE topics according to each of the 7 IPPF components is summarized in Table 4. Topics related to sexual and reproductive health knowledge were the most commonly reported: preventing sexually transmitted infections (97.7%), and HIV (97.2%), along information on the reproductive system (97.7%). The least reported topics were related to attitudes and self-efficacy on components of Sexual Rights and Citizenship (e.g How to overcome barriers to get condoms or other contraceptive methods, with less that 45%) relationships (e.g. How to talk to your partner about using condoms, 64.9%). We found significant differences between male and female students regarding exposure to certain CSE topics. Female students reported higher exposure to topics on gender-equitable attitudes (88.3% in males, 92.2% in females), on how to prevent pregnancy (95.8% in males, 97.9% in females), implications of drug and alcohol use (87.8% in males, 91.6% in females) and information on physical violence (84.0% in males 89.0%, in females).

**Table 4:** Distribution of Comprehensive Sexual Education coverage by gender, Mexico 2014.

	Male % [95%CI]	Female % [95%CI]	Total % [95%CI]
**Gender**			
Gender equity*	88.3 [86.6,89.8]	92.2 [90.8,93.4]	90.3 [89.2,91.3]
Relationships (how boys should treat girls and vice versa)	87.5 [86.0,88.9]	88.4 [86.5,90.0]	87.9 [86.7,89.1]
**Sexual and reproductive health and HIV**			
Adequate use of condoms	95.7 [94.8,96.5]	94.6 [93.6,95.4]	95.1 [94.3,95.8]
Correct use of condoms or other contraceptive methods	91.2 [89.5,92.7]	89.4 [87.8,90.8]	90.3 [88.9,91.4]
The reproductive system (where the eggs and sperm are formed and how pregnancy occurs)	97.6 [96.8,98.3]	97.7 [97.0,98.2]	97.7 [97.1,98.1]
Puberty (the way children’s bodies change during adolescence)	93.9 [92.6,95.1]	95.1 [93.4,96.4]	94.6 [93.2,95.7]
How to prevent pregnancy*	95.8 [94.9,96.5]	97.9 [97.2,98.5]	96.9 [96.4,97.4]
How to prevent HIV	97.2 [96.0,98.0]	97.3 [96.6,97.8]	97.2 [96.7,97.7]
How to prevent sexually transmitted infections	97.6 [96.4,98.4]	97.8 [97.0,98.3]	97.7 [97.1,98.1]
Implications of drug and alcohol use*	87.8 [86.0,89.3]	91.6 [90.1,92.9]	89.8 [88.6,90.8]
**Sexual rights and sexual citizenship**			
Reproductive and sexual rights	69.6 [66.5,72.6]	67.6 [64.4,70.7]	68.6 [65.8,71.2]
Access to health services	66.8 [64.6,68.9]	69.4 [67.0,71.6]	68.1 [66.1,70.1]
Where to get condoms or other contraceptive methods	81.8 [79.1,84.2]	80.8 [77.9,83.4]	81.3 [79.1,83.3]
How to overcome barriers to get condoms or other contraceptive methods	47.3 [43.8,50.9]	42.1 [38.4,45.8]	44.6 [41.5,47.7]
**Pleasure**			
Pleasure (sex should be enjoyable and not forced, sexuality is part of the life of each person, acceptance of masturbation)	69.7 [66.7,72.5]	70.4 [67.8,72.9]	70.1 [67.9,72.2]
**Violence**			
Physical violence*	84.0 [81.1,86.6]	89.0 [87.3,90.6]	86.6 [84.8,88.3]
Sexual violence	76.2 [73.4,78.9]	80.1 [77.6,82.4]	78.3 [75.9,80.4]
**Diversity**			
Respect for diversity and disability	70.7 [67.2,73.8]	74.3 [72.1,76.4]	72.6 [70.2,74.8]
Respect for people with HIV/AIDS	66.1 [63.3,68.4]	70.4 [67.1,73.5]	68.3 [65.9,70.6]
Respect for sexual orientation	66.3 [63.8,68.7]	68.7 [66.5,70.8]	67.6 [65.5,69.5]
Respect for ethnic origins	66.5 [63.5,69.3]	67.0 [64.3,69.7]	66.8 [64.4,69.1]
**Relationships**			
How to talk to your partner about using condoms	66.5 [63.8,69.1]	63.4 [60.1,66.6]	64.9 [62.0,67.7]
Values and social relations, recognition of healthy and coercive relationships	61.7 [58.2,65.0]	61.1 [57.7,64.4]	61.4 [58.4,64.2]
How to avoid situations where you could be forced to have sex or to have unprotected sex	69.8 [66.7,72.6]	73.6 [70.4,76.5]	71.7 [68.9,74.3]

* Significant at p <0.05. Difference between male and female.

Table 5 presents prevalence for sexual and reproductive health outcomes related to knowledge, attitudes and practices, organized under the 7 CSE components framework. Under the SRH component, we found a significant higher proportion of females that recognized at least three out of five effective contraceptives (98.3% in females, 95.8% in males); however, there was a higher proportion of males who knew that condoms can only be used once (88.3% in males, 75.2% in females). For the Pleasure component, we found that male students had more positive attitudes towards sex and pleasure than female students (64.8% vs 55.7% and 76.8% vs 68.0%, respectively). In the Diversity component, male students reported significantly higher prevalence of bullying compared to women, both in terms of bullying due to skin color, disability or religion (39.6% vs 29.3%) and homophobic bullying (33.4% in males, 19.2% in females). In the Relationships component, a significantly higher proportion of female students reported to be able to say that they would not want to have sex unless their partner uses a condom (56.8% in male, 76.6% in female).

**Table 5:** Distribution of outcomes related to CSE components by gender, México 2014.

	Male % [95%CI]	Female % [95%CI]	Total % [95%CI]
**Gender**			
Have at least two out of six gender-equitable attitudes	45.2 [42.4,48.1]	46.6 [43.8,49.5]	46.0 [43.8,48.1]
**Sexual and reproductive health and HIV**			
Recognize at least three out of five effective contraceptives*	95.8 [94.4,96.8]	98.3 [97.1,99.0]	97.1 [96.2,97.8]
Have correct knowledge at least five out of nine of the topics about sexual and reproductive health	88.8 [86.4,90.9]	83.4 [81.5,85.2]	86.1 [84.3,87.8]
Know that condoms can only be used once*	88.3 [86.3,90.1]	75.2 [73.5,76.8]	81.5 [80.1,82.9]
Have positive attitudes towards condoms	85.4 [83.7,87.0]	84.4 [81.2,87.1]	84.9 [83.0,86.7]
**Sexual rights and sexual citizenship**			
Know at least two out of four of their sexual and reproductive health rights	45.1 [41.3,49.0]	46.8 [43.1,50.4]	46.0 [42.5,49.5]
Sought sexual and reproductive health counseling	48.9 [45.0,52.9]	45.8 [42.6,49.0]	47.3 [44.4,50.2]
**Pleasure**			
Have positive attitudes towards sexuality*	64.8 [61.7,67.7]	55.7 [51.6,59.6]	60.1 [57.3,62.9]
Have positive attitudes towards pleasure*	76.8 [73.2,80.1]	68.0 [63.9,72.0]	73.3 [70.7,75.7]
**Violence**			
Suffered family violence over the last year	13.9 [12.4,15.5]	19.4 [17.1,21.8]	16.7 [15.2,18.4]
Suffered dating violence over the last year	22.3 [20.2,24.4]	25.3 [22.9,27.9]	23.9 [22.3,25.4]
Suffered sexual abuse (in their lifetime)	8.4 [7.1,9.8]	9.0 [7.5,10.7]	8.7 [7.7,9.8]
Suffered bullying at school over the last year*	52.8 [50.3,55.3]	43.5 [40.5,46.4]	48.0 [45.7,50.3]
**Diversity**			
During the last year, suffered bullying because of:			
The color of their skin, disability, or religion*	39.6 [36.9,42.3]	29.3 [26.3,32.5]	34.3 [31.9,36.7]
The way they express their gender	24.3 [21.7,27.2]	23.4 [21.4,25.6]	23.9 [22.2,25.6]
Homophobic bullying*	33.4 [30.9,35.9]	19.2 [17.1,21.6]	26.0 [24.5,27.7]
**Relationships**			
Make equitable decisions in their relationship	90.3 [87.3,92.7]	95.0 [91.5,97.1]	92.3 [90.3,93.9]
Can talk about using condoms with their partner	65.2 [61.6,68.7]	69.4 [66.9,71.8]	67.4 [65.0,69.7]
Can say they don’t want to have sex unless their partner uses a condom*	56.8 [52.3,61.2]	76.6 [73.8,79.2]	67.1 [63.8,70.3]
Say they could convince their partner to use condoms	54.9 [50.1,59.6]	50.9 [46.9,54.9]	52.9 [49.5,56.2]
Negotiated condom use in their first sexual encounter	19.1 [17.2,21.2]	19.7 [17.6,21.9]	19.4 [17.9,21.0]

* Significant at p <0.05. Difference between male and female.

Table 6 presents the association of exposure to CSE topics with related outcomes, adjusted for age and sex. Results show that exposure to CSE topics related to SRH component significantly increased the odds of improved SRH outcomes. More specifically, students exposed to comprehensive SRH information in school were four times more likely to recognize effective contraceptives (OR 4.10; 95% CI [2.93,5.75]; p<0.001), as well as to have positive attitudes towards condoms (OR 1.45; 95% CI [1.17,1.81]; p<0.001), and to know that condoms can only be used once (OR 1.72; 95% CI [1.40,2.12; p<0.001). Students exposed to CSE information related to Sexual Rights and Citizenship were 2.17 more likely to report having sought sexual and reproductive health counseling as compared to those who were not exposed to all the topics within this component (OR 2.17; 95% CI [1.91,2.46]; P<0.001). In the Pleasure component, students exposed to all the topics had 19% greater likelihood to have positive attitudes towards sexuality (OR 1.19; 95% CI [1.02,1.38]; p = 0.026). In the Diversity component, we found that the likelihood to suffer bullying due to skin color, a disability or religion in students exposed to comprehensive information within this component, was reduced by 20% as compared with those not exposed to all the topics in this component (OR 0.80; 95% CI [0.67,0.96]; p = 0.015). Finally, in the Relationships component students exposed to all topics were 20% more likely to affirm they could convince their partner to use condoms compared to students who were not exposed to all the topics (OR 1.20; 95% CI [1.05,1.36]; p = 0.008).

**Table 6:** Association between sexual and reproductive health outcomes and CSE coverage according to the IPPF framework.^A^ Mexico 2014.

	OR [95% CI]^B^	P value
**Gender**		
Gender-equitable attitudes	1.00 [0.75,1.34]	0.982
**Sexual and reproductive health and HIV**		
Recognize at least 3 out of five effective contraceptives	4.10 [2.93,5.75]	<0.001
Have correct knowledge on more than half of the topics about sexual and reproductive health	1.67 [1.29,2.16]	<0.001
Know that condoms can only be used once	1.72 [1.40,2.12]	<0.001
Have positive attitudes towards condoms	1.45 [1.17,1.81]	<0.001
**Sexual rights and sexual citizenship**		
Know at least half their sexual and reproductive health rights	1.13 [0.96,1.32]	0.132
Sought sexual and reproductive health counseling	2.17 [1.91,2.46]	<0.001
**Pleasure**		
Have positive attitudes towards sexuality	1.19 [1.02,1.38]	0.026
Have positive attitudes towards pleasure	1.05 [0.80,1.39]	0.698
**Violence**		
Suffered family violence during the last year	0.95 [0.79,1.15]	0.607
Suffered dating violence during the last year	0.94 [0.76,1.16]	0.556
Suffered sexual abuse (in their lifetime)	1.00 [0.76,1.31]	0.989
Suffered bullying at school over the last year	0.84 [0.70,1.01]	0.061
**Diversity**		
During the last year, suffered bullying because of:		
The color of their skin, a disability, or religion	0.80 [0.67,0.96]	0.015
The way they express their gender	0.82 [0.66,1.02]	0.079
Homophobic bullying	0.89 [0.73,1.07]	0.202
**Relationships**		
Make more than half of the decisions in their relationship	1.14 [0.60,2.17]	0.689
Can talk about using condoms with their partner	1.02 [0.88,1.19]	0.786
Can say they don't want to have sex unless their partner uses a condom	1.16 [0.99,1.37]	0.072
Say they could convince their partner to use condoms	1.20 [1.05,1.36]	0.008
Negotiated condom use in their first sexual encounter	1.08 [0.92,1.28]	0.338

A: IPPF. IPPF Framework for Comprehensive Sexuality Education. 2010.

B: Adjusted for age and sex.

## Discussion

In this study, we aimed to describe the CSE coverage and to evaluate its association with sexual and reproductive health knowledge, attitudes, and practices among a nationally representative sample of Mexican high-school students. We found that there is a high coverage on CSE topics related to biological and preventive aspects of sexual and reproductive health, however, coverage of contents more related to improving attitudes and self-efficacy is low. We also found that exposure to CSE components is significantly associated with improved knowledge of SRH, positive attitudes towards sex, and increased self-efficacy in the use of condoms and partner communication. These results add to the evidence that has shown that CSE fosters attitudes and abilities through the delivery of culturally relevant, scientifically rigorous contents that are appropriate to the stage of development of the person, thus helping to develop essential skills for making decisions related to the exercise of people’s sexuality throughout their lives, including reproductive choices [12].

We observed that exposure to CSE topics was higher in some components. Gender and SRH were the most common CSE components, with over 85% of the students reporting having reviewed them at some point during their school trajectory. In contrast, other components, such as Diversity and Relationships were reported by less than 65% of the students. It is important to note that reproductive and sexual rights, access to health services, and how to overcome barriers to obtaining condoms or other contraceptive methods were amongst the topics with the least exposure: 68.6%, 68.1%, and 44.6%, respectively.

Based on our findings, exposure to CSE components might influence attitudes, beliefs, knowledge and behaviors associated with each component. In our study, most Mexican high-school students (80–97%) had positive attitudes and basic knowledge about condoms, identified effective contraceptive methods and recognized healthy (non-coercive) relationships. Fewer had good condom use self-efficacy (53–67%), and under half had gender-equitable attitudes. In terms of practices, under half had sought sexual and reproductive health counseling (47%) and only about 20% of students negotiated condom use at first sexual encounter.

Perhaps most significant is that almost all of the explored sexual and reproductive health outcomes, which included adequate knowledge, attitudes and practices, had a positive association with full exposure to their respective CSE component. A recent meta-analysis evaluated the effects of CSE on knowledge, attitudes, and sexual risk behaviors in low- and middle-income countries. Students exposed to CSE had more knowledge about HIV and other related topics, exhibited more self-efficacy, and waited longer to initiate sexual intercourse. In addition, condom use was significantly higher among students who were exposed to CSE [10]. Our study is consistent with these findings: exposure to all the contents in SRH and HIV is associated with more knowledge about HIV related topics and contraceptive use; however, it is important to acknowledge that in some cases, such as the gender component, exposure to the component was not associated with an improved outcome, further studying the quality of CSE coverage is paramount to improve CSE programs and sexual health outcomes in adolescents.

CSE curricula has been implemented in several countries, it varies from one school to another, in the US CSE has proven to be more effective than abstinence-only programs[13]. CSE programs that achieve better results are those that extend beyond school-based sex education to include parents, health care providers and community members [10]. Evaluations of STI/HIV prevention programs for Latino and African American youth in U.S. schools found that gender roles and their impact on sexual practices were a common component in successful programs [14,15]. Our results show that exposure to the Relationships and Sexual Rights and Citizenship components were among the lowest; still, Mexican students exposed to the Relationship component had higher odds of presenting better condom negotiation skills. Another important CSE component according to the IPPF is adolescent sexual rights and citizenship [2]; in our study, being exposed to this component was associated with increased odds of seeking SRH counseling. This result is important and reflects the global call to promote an active and healthy youth engagement in society under the new SDGs agenda. In Mexico, as in other countries, adolescents have the right to access public health care, which includes SRH care, yet only a few proportion effectively exercise this right. As citizens, adolescents should be empowered to know their rights and be informed about national policies and laws that entitle them to access SRH care services. In this sense, CSE could help adolescents overcome barriers and exercise such rights, providing information about available services and resources and how to effectively access them [2].

Our results show that coverage of comprehensive sex education in schools in Mexico should be strengthened. Other Latin American countries such as Cuba or Argentina have developed successful programs by training and empowering teachers to provide adequate CSE at schools. In addition, they allowed teachers to collaborate with the government in the development of sex education programs [16,17]. Evidence shows that different types of sex education programs can be successful at improving desired outcomes for adolescents [10,18,19], yet further research is needed on effectiveness, replication and implementation studies that provide an evidence base for interventions that are effective and efficient, and how they can be implemented and scaled-up in different contexts [20].

We must acknowledge some limitations in our study, the main one being our inability to assess causality due to the cross-sectional nature of the data. Even though, we did find important associations between exposure to CSE and knowledge, attitudes, and skills; these associations warrant further study with longitudinal methods. Another important issue is the response rate; even though the schools who refused to participate were replaced by matched schools from the same area, there is a possibility of selection bias. Also, we do not have information of adolescents in schools whose parents did not consent the participation in our study. Another important limitation is that our exposure (CSE coverage) was self-reported and it is subject to recall bias, since the questionnaire asked about coverage of CSE components at any point during students’ academic career; so, if for example they were exposed to an SRH topic in elementary school they might not remember this by the time they are in high-school. Yet, there is evidence that the highest CSE exposure occurs during middle-school, and on the other hand it could be said that failure to remember CSE information should be considered as an absence of CSE coverage, so our results should not be excessively affected by this bias [11]. In addition, our exposure variable was generated considering that CSE topics had been mentioned at any given time during school years, even if it was just one time. Thus, we acknowledge that this can only be considered a *proxy* measure of the real exposure to CSE information that might have resulted in an over-estimate of coverage of CSE components. In addition, it is possible that the student might have discussed some of the topics on other contexts such as home or with friends causing misclassification of the exposure, still, in the questionnaire we specifically asked were this topic was covered (school, home, internet, friends, or other) and considered it to be covered only if they responded school (S1 and S2 Appendices).

## Conclusion

This paper provides evidence of the potential beneficial effects of CSE on attitudes, knowledge, and behaviors regarding sexual and reproductive health in adolescents in Mexico, a large middle-income country. We found important deficiencies on CSE coverage, especially in the topics designed to empower adolescents to overcome barriers and exercise their sexual and reproductive health rights. School CSE programs should be revised and improved to achieve a full coverage of all IPPF components and to work together with the community and healthcare sector to improve sexual and reproductive health outcomes in adolescents.

## Supporting information

S1 Appendix Complete questionnaire in Spanish. (DOCX)

S2 Appendix Translation of questions used in this study from the Spanish questionnaire. (DOCX)
