# Supplementary material for: Sexual and reproductive health outcomes are positively associated with comprehensive sexual education exposure in Mexican high-school students
Source: PLoS One. 2018 Mar 19;13(3):e0193780. doi: 10.1371/journal.pone.0193780 (PMC5858848; doi:10.1371/journal.pone.0193780)
Supplement: S1 Appendix — (DOCX) [file pone.0193780.s001.docx]

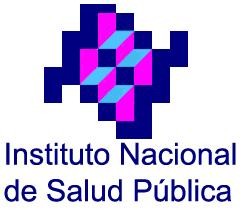


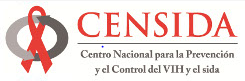
 CUESTIONARIO AUTOAPLICABLE

**CONOCIMIENTOS Y ACTITUDES EN SEXUALIDAD**

1.IDENTIFICACIÓNGEOGRÁFICA

**FAVOR DE NO CONTESTAR ESTA HOJA**

2. FOLIO

ENTIDADFEDERATIVA MUNICIPIOODELEGACIÓN LOCALIDAD

|_____|_____|_____|_____|_____|_____|_____|_____|_____|

3. CONTROL DE PAQUETE

ESTRATO

AGEB

FOLIODEPAQUETE ..............................

CONSECUTIVODELCUESTIONARIO ENELPAQUETE ....................................

4. EDUCACIÓN MEDIA SUPERIOR

CLAVE DEL CENTRO DE TRABAJO ........................................................................................... | | _| | | | | | | | |

NOMBRE DEL CENTRO DE TRABAJO: GRUPO: Centralizado **1**

Descentralizado de la federación **2**

Descentralizado de las entidades federativas **3**

Autónomo **4**

SUBGRUPO:

AGRUPACIÓN: OPCIONES EDUCATIVAS: Profesional técnico **1**

Bachillerato general: **2**

Bachillerato tecnológico **3**

5. DIRECCIÓN DE LA ESCUELA

DOMICILIO:

COLONIA:

C.P.:

TELÉFONO DE LA ESCUELA:

NOMBRE DEL(A) DIRECTOR(A):

6. DATOS DEL ALUMNO

AÑO QUE CURSA | | | CLAVE DE GRUPO | | | Matutino **…………………………………………………….1**

Vespertino **2**

Nocturno **3**

Discontinuo **4**

Mixto

I. DATOS GENERALES

LAS SIGUIENTES SON PREGUNTAS SOBRE TU FAMILIA, Y DE LA VIVIENDA DONDE HABITAS, POR FAVOR MARCA LA RESPUESTA CORRECTA.

| 1. ¿Cuántos años cumplidos tienes? | AÑOS CUMPLIDOS………………I___I___I | **Rango 15 a 18** |
| --- | --- | --- |
| 1. Indica cuál es tu sexo | Hombre …...…………………………………………  Mujer ……………………………………………...… | 1  2 |
| 1. ¿Cuál es tu estado civil? | Soltero (a) …………………………………..…….…  Casado (a) …………………………………....…..…  Separado (a) ……………………………………..…  Unión libre / vives con tu pareja…..……………..….  Divorciado (a) …………………………………….… | 1  2  3  4  5 |
| 1. ¿Cuál es tu religión?   Marca solo una opción de respuesta | Ninguna ………………………….........………….…  Católica.........…….............……………………....…  Testigo de jehova .................................................  Adventista del 7° dia …………………………….…  Evangelista ....................................................…...  Pentecostés………………………………………....  Mormón .................................................................  Otra………………………………………………...…  No quiero contestar................................................ | 1 **PASE A 1.6**  2  3  4  5  6  7  8  9 **PASE A 1.6** |
| 1. ¿Con qué frecuencia asistes a servicios religiosos? | Diario……………………………………………..….  Por lo menos una vez por semana……………….  Por lo menos una vez al mes ……………………..  Por lo menos una vez al año……………………....  Menos de una vez al año …………………………  Nunca……………………………………………….. | 1  2  3  4  5  6 |
| 1. ¿Hablas alguna lengua indígena? | Sí, y también hablo español ………………………  No hablo lengua indígena ………………………… | 1  2 |
| 1. A continuación vamos a hablar de tu ocupación principal. En la actualidad, ¿en cuál de las siguientes situaciones te encuentras? | Sólo estudio…………………………..……….……...  Principalmente estudio y hago algún trabajo…………………………………………..…..…  Principalmente trabajo y además estudio………….  Estudio y además estoy buscando trabajo…….…..  Otra situación……………………………………..….. | 1  2  3  4  5 |
| 1. ¿Cuál sería la razón por la que dejarías de seguir estudiando*?*   *Puedes contestar 2 opciones como máximo* | Para trabajar…………………………….……..….…  Para ganar dinero……………………..………..……  Para vivir mejor………………………………..….…  Para ayudar a mi familia……………….……..….…  Porque estudiar no sirve para nada…….…..…..…  No pienso dejar de estudiar………………..………  Para casarse o vivir en pareja………………...…...  Por embarazo ………………………..........…..…....  No se……………………………………………...…. | 01  02  03  04  05  06  07  08  88 |
| 1. Podrías decirme, ¿qué tan frecuentemente has faltado a clases durante el año escolar anterior? | Nunca…………………………..…….…………....…  Una vez al mes………….……….…..………..…….  Pocas veces al mes…………………………….......  Varias veces al mes…………………………..…….  Muchas veces al mes……………………..……..… | 1  PASA A 1.11  2  3  4  5 |
| 1. ¿Cuál es la principal razón por la que has faltado a clases? | Problemas de salud…………………………....…....  Problemas familiares………………………....….....  Cambio de domicilio………………………....…..….  Me expulsaron/suspendieron……………….....…...  Por el trabajo…………………………………....…...  Por irme de pinta…………………………….......….  Por embarazo ……………………………….....…...  Otra ……………………………………………....…. | 01  02  03  04  05  06  07  77 |
| 1. ¿Cuál fue el promedio general que obtuviste en el ciclo escolar pasado? | PROMEDIO………………. I___I___I . I___I |  |

**Tema: Derechohabiencia y diagnóstico previo de enfermedades**

| 1. ¿Estás afiliado(a) o inscrito(a) a los servicios médicos de …   *Selecciona hasta dos opciones* | Oportunidades………………………………………  IMSS………………………….………………..…..…  ISSSTE.…………………….…………….………...  SECMAR.…………………….……….……..……..  SEDENA.…………………….………..…………..…  PEMEX.………………….………………………....  SEGURO POPULAR.…….……………..........……  SEGURO PRIVADO..............................................  No…………………………………………………….  No se…………………………………………….… | 01  02  03  04  05  06  07  08  09  88 |
| --- | --- | --- |
| 1. Cuando tienes problemas de salud ¿en dónde te atiendes usualmente? | Oportunidades…………………………………….…  IMSS………………………….………………..…..…  ISSSTE.…………………….…………….……….....  SECMAR.…………………….……….……..……....  SEDENA.…………………….………..…………..…  PEMEX.………………….…………………………..  SEGURO POPULAR.…….……………..........……  SEGURO PRIVADO..............................................  Consultorio asociado a alguna Farmacia……..…  No lo sé…………………………………………...… | 01  02  03  04  05  06  07  08  09  88 |
| 1. ¿Algún médico te ha diagnosticado…   Puedes marcar más de una opción | asma?....................................................................  tuberculosis?.........................................................  presión alta (hipertensión)? ………………….........  diabetes (azúcar alta en la sangre)?.....................  enfermedad del corazón?………..………….........  fiebre reumática?...................................................  enfermedad renal? …………………………………  depresión? ……………..……………………………  psicosis? …………………………………………..  anorexia o bulimia? …………………………….…  gastritis o úlcera gástrica?…………………………  colitis?...................................................................  tumores, cáncer? ……………………....…………..  VIH y/ o sida ?........................................................  obesidad/sobrepeso?………………………………  problemas de aprendizaje?…………………….…  hiperactividad y/o déficit de atención?………….  problemas conductuales?………………………..  Otra enfermedad con duración mayor a 3 meses ……………………………………………………….  NINGUNA……………………………..................... | 01  02  03  04  05  06  07  08  09  10  11  12  13  14  15  16  17  18  19  **20** |

**II. CARACTERÍSTICAS DE LA VIVIENDA**

**Las siguientes preguntas se refieren a las características de tu casa y los servicios con los que cuenta.**

| 1. ¿Cuántos cuartos se usan para dormir sin contar pasillos? | \|___\|___\|  Cuartos dormitorio | **Rango 0 a 10** |
| --- | --- | --- |
| 1. ¿Cuántas personas viven en tu vivienda? | \|___\|___\| |  |
| 1. ¿La vivienda donde tu vives es… | propia (de alguno de tus padres es el dueño)?......  prestada?................................................................  rentada? .................................................................  Otro…………………………………………………… | 1  2  3  7 |

**Las siguientes preguntas refieren a los bienes que son de tu propiedad o de alguno de los miembros de tu hogar.**

| 1. **¿Tu o algún integrante de tu hogar (que viva en tu casa) tienen.....** | | | |
| --- | --- | --- | --- |
|  | **BIENES DEL HOGAR** | **SI** | **NO** |
| 01 | automóvil? | 1 | 2 |
| 02 | televisión? | 1 | 2 |
| 03 | servicio de TV de paga? (cablevisión, sky, etc.) | 1 | 2 |
| 04 | computadora de escritorio o laptop? | 1 | 2 |
| 05 | servicio de Internet? | 1 | 2 |
| 06 | horno de microondas? | 1 | 2 |
| 07 | línea telefónica fija? | 1 | 2 |

**III. DATOS FAMILIARES**

| **EN ESTA SECCIÓN ENCONTRARÁS PREGUNTAS ACERCA DE TU FAMILIA POR FAVOR SELECCIONA TU RESPUESTA.** | | | |
| --- | --- | --- | --- |
| 3.1 ¿Con quién vives?  PUEDES MARCAR MÁS DE UNA OPCIÓN | Padre……………………………………………...…...  Madre………………………….…………………....…  Los dos son padres…………………………………  Las dos son madres…………………………………  Hermanos (as)……………………………..……...….  Otros familiares…………………………………...….  Otra personas que no son familiares…………...….  Solo (a)……………………………………….…….....  Con amigos………………………………………...…  Casa de asistencia…………………….………...….. | 1  2  3  4  5  6  7  8  9  10 |  |
| 3.2 ¿Tus padres actualmente están… | casados y viven juntos?...............................…...….  viven juntos pero no se han casado?...........…...…  casados pero viven separados?..................…...….  divorciados?..................................................…...…  Mamá es viuda………………………………..…...…  Papá es viudo………………………….……..…...…  Los dos han muerto………………..……………...…  Mamá es soltera…………………………………...…  Padre es soltero…………………………………...… | 1  2  3  4  5  6  7  8  9 |  |
| 3.3 ¿Cuál es el nivel escolar que alcanzó tu papá? | Sin escolaridad.…………………………………...….  Primaria incompleta…………….….……………...…  Primaria completa………………….…………...…….  Secundaria incompleta……………….….…...…..….  Secundaria completa………………………...………  Preparatoria incompleta…………………..…...……  Preparatoria completa………………………...…..…  Técnico…………………………………………...…...  Profesional………………………………………...….  Posgrado………………………………………...……  No tengo papá…………………………..……........…  No lo sé………………………………….........…........ | 01  02  03  04  05  06  07  08  09  10  11  88 |  |
| 3.4 ¿Cuál es el nivel escolar que alcanzó tu mamá? | Sin escolaridad.…………………………………...….  Primaria incompleta…………….….……………...…  Primaria completa………………….…………...…….  Secundaria incompleta……………….….…...…..….  Secundaria completa………………………...………  Preparatoria incompleta…………………..…...……  Preparatoria completa………………………...…..…  Técnico…………………………………………...…...  Profesional………………………………………...….  Posgrado………………………………………...……  No tengo mamá…………………………..……........  No lo sé………………………………….........…........ | 01  02  03  04  05  06  07  08  09  10  11  88 |  |

**IV. RELACIONES ENTRE IGUALES EN LA ESCUELA Y EN TIEMPO LIBRE**

**Las siguientes preguntas que vas a contestar están relacionadas con el trato emocional que recibiste durante el año pasado.**

| - 1. ¿Con que frecuencia durante el último año (12 meses) en tu casa o en la escuela ………   **PASA A SIGUENTE INCISO**  Nunca o casi nunca……………..…….1  Algunas veces………………………….2  Frecuentemente  (2 veces en una semana) ……………..3  Muy frecuentemente  (más de 2 veces en una semana) …….4  **No quiero contestar …………………… 5 (pasar a 4.4)** | | - 1. Quién te ha (MENCIONAR RESPUESTA DE **4.1**)?   Padre………..1  Madre……….2  Hermanos..…3  Compañeros..4  Profesores….5  Otros……..…7 | - 1. ¿En qué lugar (MENCIONAR RESPUESTA DE **4.1**)?   Casa…………….1  Escuela…………2  Ambos…………3 |
| --- | --- | --- | --- |
| A) Te dicen insultos o groserías?.................................. | [__] | [__] | [__] |
| B) Te hacen sentir mal?........................................... | [__] | [__] | [__] |
| C) Te provocan o te hacen sentir miedo?.................... | [__] | [__] | [__] |
| D) Te han cacheteado, pegado o golpeado?.................. | [__] | [__] | [__] |

| - 1. Marca qué tanto las siguientes cosas son ciertas para ti: | Nunca | De vez en cuando | Bastantes veces | Muchas veces | **No quiero contestar** |
| --- | --- | --- | --- | --- | --- |
| A) Me dicen nombres feos. | 0 | 1 | 2 | 3 | **4** |
| B) Me molestan o me agreden. | 0 | 1 | 2 | 3 | **4** |
| C) Me gusta molestar a los debiluchos. | 0 | 1 | 2 | 3 | **4** |
| D) Me gusta pelearme con alguien a quien le puedo ganar fácilmente | 0 | 1 | 2 | 3 | **4** |
| E) Se burlan de mi. | 0 | 1 | 2 | 3 | **4** |
| F) Me pegan o me dan empujones. | 0 | 1 | 2 | 3 | **4** |
| G) Soy parte de un grupo que se burla de otras personas. | 0 | 1 | 2 | 3 | **4** |
| H) Me gusta hacer que otras personas me tengan miedo. | 0 | 1 | 2 | 3 | **4** |

**Bullying por orientación sexual y otros motivos**

| - 1. ¿Qué tan frecuentemente escuchas expresiones como “qué gay es eso”, “eres muy gay”, en la escuela? | Muchísimas veces……………………………….....  Con frecuencia …………………………………..…  Algunas veces …………………………………..….  Raras veces ……………………………………..….  Nunca……………………………………………..….  **No quiero contestar ………………………………** | 1  2  3  4  5  **6** |
| --- | --- | --- |
| - 1. ¿Qué tan frecuentemente has oído otras expresiones como “puto”, “marica” o “lesbiana” usada de manera insultante, en la escuela? | Muchísimas veces……………………………….....  Con frecuencia …………………………………..…  Algunas veces …………………………………..….  Raras veces ……………………………………..….  Nunca……………………………………………..….  **No quiero contestar ………………………………** | 1  2  3  4  5  **6** |
| - 1. Dirías que en la escuela, estas expresiones las usan: | La mayoría de los estudiantes ……………………  Algunos de los estudiantes………………………..  Pocos de los estudiantes………………………..…  **No quiero contestar ………………………………** | 1  2  3  **4** |
| - 1. ¿Te sientes inseguro/a en la escuela por:   *Marca todas las que aplican para ti* | a) tu orientación sexual?.....................................  b) tu género (por ser mujer o varón)?..................  c) tu color de piel o grupo étnico? ………………  d) la manera en que expresas tu género (qué tan tradicionalmente “masculino” o “femenina” eres en cómo te ves o tu manera de actuar)?......  e) una discapacidad que tengas o que las personas creen que tengas?................................  f) tu religión o porque la gente cree que eres de una cierta religión?...............................................  g) No me siento inseguro…………………………..  **No quiero contestar ………………………………** | 1  2  3  4  5  6  7  **8** |

| - 1. En el último año, qué tan frecuentemente te han molestado verbalmente (llamándote palabras feas, amenazas, etc.) en tu escuela por: | Muchísimas veces | Con frecuencia | Algunas veces | Raras veces | Nunca | **No quiero contestar** |
| --- | --- | --- | --- | --- | --- | --- |
| a) tu orientación sexual? | 4 | 3 | 2 | 1 | 0 | **5** |
| b) tu género (por ser mujer o varón)? | 4 | 3 | 2 | 1 | 0 | **5** |
| c) tu color de piel o grupo étnico? | 4 | 3 | 2 | 1 | 0 | **5** |
| d) la manera en que expresas tu género (qué tan tradicionalmente “masculino” o “femenina” eres en cómo te ves o tu manera de actuar)? | 4 | 3 | 2 | 1 | 0 | **5** |
| e) una discapacidad que tengas o que las personas creen que tengas? | 4 | 3 | 2 | 1 | 0 | **5** |
| f) tu religión o porque la gente cree que eres de una cierta religión? | 4 | 3 | 2 | 1 | 0 | **5** |
| - 1. En el último año, qué tan frecuentemente te han molestado físicamente (empujado, pegado, etc.) en tu escuela por: | Muchísimas veces | Con frecuencia | Algunas veces | Raras veces | Nunca | **No quiero contestar** |
| a) tu orientación sexual? | 4 | 3 | 2 | 1 | 0 | **5** |
| b) tu género (por ser mujer o varón)? | 4 | 3 | 2 | 1 | 0 | **5** |
| c) tu color de piel o grupo étnico? | 4 | 3 | 2 | 1 | 0 | **5** |
| d) la manera en que expresas tu género (qué tan tradicionalmente “masculino” o “femenina” eres en cómo te ves o tu manera de actuar)? | 4 | 3 | 2 | 1 | 0 | **5** |
| e) una discapacidad que tengas o que las personas creen que tengas? | 4 | 3 | 2 | 1 | 0 | **5** |
| f) tu religión o porque la gente cree que eres de una cierta religión? | 4 | 3 | 2 | 1 | 0 | **5** |

**ESCUELAS**

| **Las siguientes preguntas corresponden a la educación que recibiste en la primaria, secundaria o preparatoria. Por favor marca tus respuestas.** | | | | | | | |
| --- | --- | --- | --- | --- | --- | --- | --- |
| - 1. ¿Durante la primaria, secundaria o preparatoria, en alguna ocasión algún maestro, orientador o el director les habló sobre:….   Sí, un(a) maestro(a)…..….1  Sí, un(a) orientador(a)…...2  Sí el(la) director(a)………..3  PASA A SIGUIENTE INCISO  No…………………….…..4  **No quiero contestar ……..5 (pasar a 4.16)** | | - 1. ¿Cuándo te hablaron de _________?   (PUEDES MARCAR UNO O MÁS)  En la primaria................1  Secundaria....................2  Preparatoria..................3 | | | - 1. Respecto a la última vez que trataron el tema en la escuela ¿Cómo fue el método de enseñanza?   (PUEDES MARCAR UNO O MÁS MÉTODOS)  Presentación por parte del maestro...........1  Ejercicios/talleres didácticos.......................2  Debate, foro o discusión en grupo..............3  Panel, mesa redonda…..............................4  Juegos y simulaciones, juego de roles.......5  Lectura de autoaprendizaje o leer tú solo...6  Búsqueda de información………….............7  Resolución de ejercicios..............................8 | | |
|  | | OPCIÓN1 | OPCIÓN2 | OPCIÓN3 | OPCIÓN1 | OPCIÓN2 | OPCIÓN3 |
| a) La pubertad (la forma en la que el cuerpo de los niños y niñas cambia durante la adolescencia) | I___I | I___I | I___I | I___I | I___I | I___I | I___I |
| b) El sistema reproductivo (en dónde se forman los óvulos y espermatozoides y cómo ocurre un embarazo) | I___I | I___I | I___I | I___I | I___I | I___I | I___I |
| c) Las relaciones (Cómo los niños deberían tratar a las niñas y viceversa) | I___I | I___I | I___I | I___I | I___I | I___I | I___I |
| d) Cómo protegerte para prevenir el VIH | I___I | I___I | I___I | I___I | I___I | I___I | I___I |
| e) Cómo protegerte para prevenir otras infecciones de transmisión sexual | I___I | I___I | I___I | I___I | I___I | I___I | I___I |
| f) Cómo protegerte para prevenir un embarazo | I___I | I___I | I___I | I___I | I___I | I___I | I___I |
| g) El uso del condón | I___I | I___I | I___I | I___I | I___I | I___I | I___I |
| h) Cómo hablar con tu pareja sobre usar condón | I___I | I___I | I___I | I___I | I___I | I___I | I___I |
| i) Como evitar situaciones que pudieran llevarte a tener relaciones sexuales que no deseas o sin protección | I___I | I___I | I___I | I___I | I___I | I___I | I___I |
| j) El uso correcto del condón o de otros métodos anticonceptivos | I___I | I___I | I___I | I___I | I___I | I___I | I___I |
| k) Dónde conseguir condones u otros métodos anticonceptivos | I___I | I___I | I___I | I___I | I___I | I___I | I___I |
| l) Como vencer barreras para conseguir condones u otros métodos  anticonceptivos | I___I | I___I | I___I | I___I | I___I | I___I | I___I |
| m) Igualdad o equidad de género | I___I | I___I | I___I | I___I | I___I | I___I | I___I |
| n) Los derechos sexuales y reproductivos | I___I | I___I | I___I | I___I | I___I | I___I | I___I |
| o) Violencia física | I___I | I___I | I___I | I___I | I___I | I___I | I___I |
| p) Violencia sexual | I___I | I___I | I___I | I___I | I___I | I___I | I___I |
| q) El placer (que el sexo debe ser agradable y no forzado, sexualidad como parte de la vida de cada persona, aceptación de la masturbación) | I___I | I___I | I___I | I___I | I___I | I___I | I___I |
| r) Implicaciones del uso de alcohol y drogas | I___I | I___I | I___I | I___I | I___I | I___I | I___I |
| s) Valores y relaciones sociales, reconocimientos de las relaciones saludables y coercitivas | I___I | I___I | I___I | I___I | I___I | I___I | I___I |
| t) Respeto de la diversidad por origen  étnico | I___I | I___I | I___I | I___I | I___I | I___I | I___I |
| u) Respeto de la diversidad por orientación sexual | I___I | I___I | I___I | I___I | I___I | I___I | I___I |
| v) Respeto de la diversidad por estatus de VIH y sida | I___I | I___I | I___I | I___I | I___I | I___I | I___I |
| x) Respeto de la diversidad por discapacidad | I___I | I___I | I___I | I___I | I___I | I___I | I___I |
| y) Acceso a servicios de salud | I___I | I___I | I___I | I___I | I___I | I___I | I___I |

**En preguntas 4.14 y 4.15, para cada respuesta afirmativa (PREG 4.11), preguntar:**

| - 1. ¿En general qué tan satisfecho te sientes con… | **Nada**  **Satisfecho** | |  | **Completamente**  **Satisfecho Satisfecho** | | |  |
| --- | --- | --- | --- | --- | --- | --- | --- |
| a) los puntos tratados | 0 | 1 | 2 | 3 | 4 | 5 |  |
| b) la intensidad con la que se abordó el tema | 0 | 1 | 2 | 3 | 4 | 5 |  |
| c) el formato empleado para abordar el tema | 0 | 1 | 2 | 3 | 4 | 5 |  |
| d) la claridad con la que se presentó el tema | 0 | 1 | 2 | 3 | 4 | 5 |  |

|  | - 1. Te pareció que el maestro fue..... | **SI** | **NO** |
| --- | --- | --- | --- |
| 01 | ¿Incluyente? | 1 | 2 |
| 02 | ¿Accesible? | 1 | 2 |
| 03 | ¿Te dio confianza? | 1 | 2 |
| 04 | ¿Crees que guardaría confidencialidad? | 1 | 2 |
| 05 | ¿Prejuicioso? | 1 | 2 |
| 06 | ¿Penoso? | 1 | 2 |
| 07 | ¿Burlón? | 1 | 2 |

| - 1. ¿Te gustaría que en la escuela trataran algún otro tema? | Sí, ¿Cuál?______________________________  ESPECIFICA  No………………………………………………….2 |  |
| --- | --- | --- |

V. RELACIÓN DE NOVIAZGO

En esta sección encontrarás preguntas sobre cómo percibes las relaciones de noviazgo o con parejas sexuales o emocionales (hayas tenido o no novio/a antes) y sobre algunas situaciones que han podido suceder durante tu última o actual relación. Por favor selecciona tu respuesta.

| 1. ¿Qué harías en caso de que tu novio(a)/pareja te insulte, amenace, ridiculice, se burle de ti, te prohíba tener amigos(as)?   PUEDES **SELECCIONAR** MAS DE UNA OPCIÓN | Lo(a) dejaría .........................................................  Lo(a) perdonaría y seguiría con él(ella) …..........  Yo le haría lo mismo .............................................  Se lo contaría a mi mejor amigo(a) .......................  Se lo contaría a algún familiar ..............................  Se lo contaría a algún maestro ............................  Pediría ayuda a un centro de atención ................  No le contaría a nadie ...........................................  Trataría de resolver la situación ..........................  Otro …………………………………………….........  **No quiero contestar ………………………………** | 01  02  03  04  05  06  07  08  09  77  **99** |
| --- | --- | --- |
| 1. ¿Qué harías tú en caso de que tu novio(a) te agrediera físicamente?   PUEDES **SELECCIONAR** MAS DE UNA OPCIÓN | Lo(a) dejaría .........................................................  Lo(a) perdonaría y seguiría con él(ella) …..........  Yo le haría lo mismo .............................................  Se lo contaría a mi mejor amigo(a) .......................  Se lo contaría a algún familiar ..............................  Se lo contaría a algún maestro ............................  Pediría ayuda a un centro de atención ................  No le contaría a nadie ...........................................  Trataría de resolver la situación ..........................  Otro …………………………………………….........  **No quiero contestar ………………………………** | 01  02  03  04  05  06  07  08  09  77  **99** |
| 1. ¿Qué harías tú en caso de que tu novio(a) te obligara a tener relaciones sexuales en contra de tu voluntad?   PUEDES **SELECCIONAR** MAS DE UNA OPCIÓN | Lo(a) dejaría .........................................................  Lo(a) perdonaría y seguiría con él(ella) …..........  Yo le haría lo mismo .............................................  Se lo contaría a mi mejor amigo(a) .......................  Se lo contaría a algún familiar ..............................  Se lo contaría a algún maestro ............................  Pediría ayuda a un centro de atención ................  No le contaría a nadie ...........................................  Trataría de resolver la situación ..........................  Otro ……………………………………………........  **No quiero contestar ………………………………** | 01  02  03  04  05  06  07  08  09  77  **99** |
| 1. Durante los últimos 12 meses, ¿Cuántas veces tu novio/a o alguien con quien estabas saliendo:   …te insultó, dijo que eras tonto/a, te ridiculizó, se burló de ti, te prohibió tener amigos o extendió rumores falsos sobre ti? | No salí con nadie o no tuve novio en los últimos 12 meses………………………………………….….  0 veces…………………………………….….……...  1 vez……………………………………….….……...  2 o 3 veces……………………………….….………  4 o 5 veces……………………………….….………   1. o más veces…………………………….…..…..…   **No quiero contestar ………………………………** | PASA A 5.7  1  2  3  4  5  6 |
| 1. Durante los últimos 12 meses, ¿Cuántas veces tu novio/a o alguien con quien estabas saliendo:   ….te lastimó físicamente a propósito?  (Incluye cosas como que te haya pegado, aventado y te pegaste en algo, lastimado con un objeto o un arma.) | No salí con nadie o no tuve novio en los últimos 12 meses………………………………………….….  0 veces…………………………………….….……...  1 vez……………………………………….….……...  2 o 3 veces……………………………….….………  4 o 5 veces……………………………….….………   1. o más veces…………………………….…..…..…   **No quiero contestar ………………………………** | 1  2  3  4  5  6  **7** |
| 1. Durante los últimos 12 meses, ¿Cuántas veces tu novio/a o alguien con quien estabas saliendo:   … te forzó a hacer cosas sexuales que no querías hacer? (Incluye cosas como besarse, tocar o que te haya forzado físicamente a tener relaciones sexuales.) | No salí con nadie o no tuve novio en los últimos 12 meses………………………………………….….  0 veces…………………………………….….……...  1 vez……………………………………….….……...  2 o 3 veces……………………………….….………  4 o 5 veces……………………………….….………   1. o más veces…………………………….…..…   **No quiero contestar ………………………………** | 1  2  3  4  5  6  **7** |
| 1. ¿Tienes o has tenido novio(a)? | Novio……………………………………….….……..  Novia……………………………………….….……..  No he tenido…………………………………………  **No quiero contestar ………………………………** | 1  2  PASA A 5.9  3  **4 (pasar a 5.9)** |

| 1. En tu relación de noviazgo actual o última relación de noviazgo, ¿Quién decide (decidía) sobre los siguientes aspectos… | **Yo** | **Él (ella)** | **Ambos** |
| --- | --- | --- | --- |
| c) Cuándo visitar a los amigos | 1 | 2 | 3 |
| d) Participar en actividades escolares | 1 | 2 | 3 |
| e) Qué tipo de ropa debes usar | 1 | 2 | 3 |
| f) Cuándo ir al cine o salir de paseo | 1 | 2 | 3 |
| g) Cuándo ir al antro o fiestas | 1 | 2 | 3 |
| h) Cuándo faltar a una clase o cuándo irte de pinta | 1 | 2 | 3 |

| 1. ¿Quién te atrae sexualmente? | Hombres……………………………………..……….  Mujeres……………………………………….………  Hombres y mujeres.…………………………..…….  Todavía no siento atracción sexual por nadie……  **No quiero contestar ………………………………** | 1  2  3  4  **5** |
| --- | --- | --- |
| 1. ¿Cómo describirías tu orientación sexual? | Heterosexual (atraído/a sexualmente al sexo opuesto)……………………………………………...  Principalmente heterosexual……………………....  Bisexual (atraído/a sexualmente a hombres y mujeres)……………………………………………...  Gay, lesbiana u homosexual (atraído al mismo sexo)………………………………………………….  No estoy seguro/a todavía……………………..….  No entiendo la pregunta………………………..…..  **No quiero contestar ………………………………** | 1  2  3  4  5  6  **7** |
| 1. Cuando piensas en o tienes fantasías sobre tener relaciones sexuales, ¿piensas en… | Hombres?..............................................................  Mujeres? ...............................................................  Hombres y mujeres?.............................................  No tengo fantasías sobre las relaciones sexuales. …..........................................................  **No quiero contestar ………………………………** | 1  2  3  4  **5** |

VI. ACTITUDES Y CONOCIMIENTOS DE CONDÓN Y MÉTODOS ANTICONCEPTIVOS

A continuación nos referiremos a los métodos o medios que puede usar una pareja para evitar o planear un embarazo.

| 1. ¿Has oído hablar de (METODO)…   Sí ………………………..………………..1  PASA A SIGUIENTE INCISO  No……………………………………..….2  **No quiero contestar …………………….3 (pasar a 6.4)** | | 1. ¿Conoces algún lugar o persona donde los ADOLESCENTES puedan obtener (MÉTODO DEL INCISO DE 6.1)?   Sí……………….………..1  No……………………….2 | 1. ¿Dónde escuchaste de la existencia de (MÉTODO DEL INCISO DE 6.1)?   Escuela ................................. 1  Casa ...................................... 2  Institución pública  o privada ............................... 3  Internet .................................. 4  Amigos .................................. 5  Otro lugar……………………….7 |
| --- | --- | --- | --- |
| a) las pastillas o píldoras anticonceptivas? | I___I | I___I | I___I |
| b) las inyecciones hormonales para anticoncepción? | I___I | I___I | I___I |
| c) del condón? | I___I | I___I | I___I |
| d) la pastilla de anticoncepción de emergencia? (del día después) | I___I | I___I | I___I |

| 1. Existen otros métodos que no se mencionaron, ¿qué otros métodos has escuchado hablar?   PUEDES **SELECCIONAR** MAS DE UNA OPCIÓN | Retiro………………………………………………....  Ritmo/abstinencia periódica……………………..…  Pastillas…………………………………………..…..  Inyección…………………………………………...…  Condón…………………………………………….....  Pastillas de emergencia……………………….…....  DIU……………………………………………….…...  Parche………………………………………….…..…  Espumas u óvulos.…………………………..………  Esterilización femenina………………………….….  Esterilización masculina…………………………....  Otros…………………………………………………..  **No quiero contestar ………………………………** | 01  02  03  04  05  06  07  08  09  10  11  77  **99** |
| --- | --- | --- |
| 1. ¿Cuál método consideras que es más adecuado para ser utilizado por un(a) adolescente?   PUEDES **SELECCIONAR** MAS DE UNA OPCIÓN | Retiro………………………………………………....  Ritmo/abstinencia periódica……………………..…  Pastillas…………………………………………..…..  Inyección…………………………………………...…  Condón…………………………………………….....  Pastillas de emergencia……………………….…....  DIU……………………………………………….…...  Parche………………………………………….…..…  Espumas u óvulos.…………………………..………  Otro……………………………………..………….….  No se……………………………………..……….…..  **No quiero contestar ………………………………** | 01  02  03  04  05  06  07  08  09  77  88  **99** |
| 1. ¿Crees que algún método podría dañar tu salud?   PUEDES **SELECCIONAR** MAS DE UNA OPCIÓN | Retiro………………………………………………....  Ritmo/abstinencia periódica……………………..…  Pastillas…………………………………………..…..  Inyección…………………………………………...…  Condón…………………………………………….....  Pastillas de emergencia……………………….…....  DIU……………………………………………….…...  Parche………………………………………….…..…  Espumas u óvulos.…………………………..………  NINGUNO ……………………………………………  Otro……………………………………………..….….  No se…………………………………………..….…..  **No quiero contestar ………………………………** | 01  02  03  04  05  06  07  08  09  10  77  88  **99** |
| 1. ¿Cuántas veces se puede usar un condón masculino? | I___I veces  No sé.....................................8 |  |
| 1. El condón masculino se utiliza como un método para prevenir un embarazo o como un método para prevenir una infección de transmisión sexual? | Para prevenir un embarazo....................................  Para prevenir una infección de transmisión sexual......................................................................  Para ambos.............................................................  No lo sé...................................................................  **No quiero contestar ………………………………** | 1  2  3  8  **9** |
| 1. ¿Alguna vez has visto un condón? | Sí………………………………………..………….....  No…………………………………………..……….... | 1  2 |
| 1. ¿En qué consiste la anticoncepción de emergencia?   **En tomar una o varias pastillas anticonceptivas lo más pronto posible pero máximo…** | …en las primeras 24 hrs. después de tener una relación sexual no protegida ………………………  …en las primeras 120 hrs. después de tener una relación sexual no protegida ………………………  …en el primer mes. después de tener una relación sexual no protegida ………………………  No se ……………………..………………………….  **No quiero contestar ………………………………** | 1  2  3  8  **99** |
| 1. ¿Cuáles de los siguientes métodos crees que sirvan para prevenir una infección de transmisión sexual o VIH y/ o sida   PUEDES MARCAR MAS DE UNA OPCIÓN | Retiro o venirse afuera? ……..………………….....  Pastillas o píldoras? ……..……..……..………...….  Lavado vaginal? ……………………………….....…  Condones? …………………………….…..……..….  Ritmo o calendario? …………………………..….…  Tomar té de hierbas? ………………………..……..  Relaciones sólo con tu pareja? ……..…………….  No tener relaciones sexuales?…………..…….......  Otro?.......................................................…….......  No quiero contestar………………...…………..…... | 01  02  03  04  05  06  07  08  77  99 |

**Conocimiento y actitudes del uso del condón**

Las personas pueden tener diferentes opiniones sobre el tema del condón.

| 1. Que tan de acuerdo o en desacuerdo estas con las siguientes opiniones | De acuerdo | Ni de acuerdo  ni en desacuerdo | En desacuerdo | **No quiero contestar** |
| --- | --- | --- | --- | --- |
| a) Los condones son un método efectivo para prevenir embarazos | 1 | 2 | 3 | **4** |
| b) Los condones pueden ocuparse más de una vez | 1 | 2 | 3 | **4** |
| c) Una mujer puede pedir a su novio que utilice condón | 1 | 2 | 3 | **4** |
| d) Un hombre puede pedir a su novia que utilice condón | 1 | 2 | 3 | **4** |
| e) Los condones son una manera efectiva de protegerse contra el VIH y/ o sida | 1 | 2 | 3 | **4** |
| f) El condón es adecuado para las relaciones ocasionales | 1 | 2 | 3 | **4** |
| g) El condón es adecuado para las relaciones amorosas estables | 1 | 2 | 3 | **4** |
| h) Sería muy vergonzoso para alguien como yo comprar u obtener condones | 1 | 2 | 3 | **4** |
| i) Si una adolescente sugiere el uso del condón a su pareja significa que ella no confía en él | 1 | 2 | 3 | **4** |
| j) Los condones reducen el placer sexual | 1 | 2 | 3 | **4** |
| k) Los condones pueden zafarse del pene y desaparecer dentro del cuerpo de la mujer | 1 | 2 | 3 | **4** |
| l) Si una pareja no está casada y desea tener relaciones sexuales antes del matrimonio, debería de usar condón | 1 | 2 | 3 | **4** |
| m) Los condones son una manera efectiva de protegerse contra enfermedades de transmisión sexual | 1 | 2 | 3 | **4** |

**Auto-eficacia del condón**

| A continuación se te presentan algunas situaciones, que muestra qué tan seguro/a estás de que pudieras hacer las siguientes cosas. No hay respuestas correctas ni incorrectas. | | | | | | |
| --- | --- | --- | --- | --- | --- | --- |
| 1. **¿Qué tan seguro(a) estas de que podrías…** | Muy inseguro(a) | Inseguro(a) | Algo seguro(a) | Seguro(a) | Muy seguro(a) | **No quiero contestar** |
| 1. Traer un condón conmigo en caso de necesitar uno. | 1 | 2 | 3 | 4 | 5 | **6** |
| 1. Hablar sobre usar condones con cualquier pareja sexual. | 1 | 2 | 3 | 4 | 5 | **6** |
| 1. Hablar sobre usar un condón si no estuviera seguro/a de los sentimientos de mi pareja sobre los condones. | 1 | 2 | 3 | 4 | 5 | **6** |
| 1. Hablar sobre usar un condón con una pareja potencial antes de que empezáramos a abrazarnos y besarnos. | 1 | 2 | 3 | 4 | 5 | **6** |
| 1. Convencer a una pareja de usar un condón cuando tengamos relaciones sexuales. | 1 | 2 | 3 | 4 | 5 | **6** |
| 1. Decir que no quiero tener relaciones sexuales si mi pareja no quisiera usar un condón. | 1 | 2 | 3 | 4 | 5 | **6** |
| 1. Usar un condón cada vez que tuviera relaciones sexuales con mi pareja. | 1 | 2 | 3 | 4 | 5 | **6** |
| 1. Usar un condón nuevo cada vez que tuviera relaciones sexuales con mi pareja. | 1 | 2 | 3 | 4 | 5 | **6** |
| 1. Detenerme y poner un condón a mí mismo o a mi pareja. | 1 | 2 | 3 | 4 | 5 | **6** |
| **¿Qué tan seguro(a) estas de que…** |  |  |  |  |  |  |
| 1. Yo o mi pareja podría enrollar un condón hasta la base del pene. | 1 | 2 | 3 | 4 | 5 | **6** |
| 1. Yo o mi pareja podría usar un condón sin que se zafara o se resbalara. | 1 | 2 | 3 | 4 | 5 | **6** |
| 1. Yo o mi pareja podría deshacernos de un condón en la basura después de las relaciones sexuales. | 1 | 2 | 3 | 4 | 5 | **6** |
| 1. Yo o mi pareja podría detener el condón en la base del pene al retirar el pene después de las relaciones sexuales. | 1 | 2 | 3 | 4 | 5 | **6** |
| 1. Usar un condón si tomara cerveza, vino u otra bebida de alcohol. | 1 | 2 | 3 | 4 | 5 | **6** |

**INICIO DE VIDA SEXUAL**

Las siguientes preguntas están relacionadas con tu experiencia sexual, recuerda que tus respuestas son estrictamente confidenciales

| 1. ¿Has tenido alguna vez relaciones sexuales?   (Ya sea penetración vaginal o anal) | Sí penetración vaginal…………..…….....………...  Sí penetración anal…………..……....……..…….....  Sí, ambas..…….....…….....…….....……......……...  No…………………………………………....……....  **No quiero contestar ………………………………** | 1  2  3  4 PASA A 6.48  **5** |
| --- | --- | --- |
| 1. ¿A qué edad tuviste tu primera relación sexual?   (Ya sea penetración vaginal o anal) | EDAD……………………………I___I___I  No me acuerdo…………………………………...  **No quiero contestar ………………………………** | 87 |
| 1. ¿Con quién tuviste tu primera relación sexual? | Con mi novio……………………………………..…..  Con mi novia……………………………………..…..  Con un amigo…………………………………..…....  Con una amiga……………………………………....  Con un familiar……………………………………....  Con un trabajador(a) sexual/prostituto(a).….……..  Con un maestro(a).………………………….……....  Con mi esposo(a)……………………….…..……....  Con un desconocido…………………….…..……...  Con una desconocida……………………….……....  Con otra persona…………………………………....  **No quiero contestar ………………………………** | 01  02  03  04  05  06  07  08  09  10  11  **99** |
| 1. ¿Qué edad tenia tú pareja de esa primera relación sexual? | EDAD……………………………I___I___I  No me acuerdo……………………………………..  No se……………………………………………...….  **No quiero contestar ………………………………** | 87  88  **99** |
| 1. ¿Por qué tuviste tu primera relación sexual? | Por amor……………………………………………..  Porque tenía curiosidad…………………….……...  Porque mi pareja me quería………………………..  Porque tenía deseo (ganas)……………...………..  Porque me forzaron………………….…..………...  Porque tuve miedo de perder a mi pareja……..…  Otra razón............……….....…………..………..…  **No quiero contestar ………………………………** | 1  2  3  4  5  6  7  **8** |
| 1. ¿Cuándo tuviste esa primera relación sexual, habías tomado alcohol? | Sí…………………………………………………......  No………………………………………………….....  **No quiero contestar ………………………………** | 1  2  **3** |
| 1. ¿Hablaste con alguien sobre qué método usar antes de la primera relación sexual?   PUEDES MARCAR MAS DE UNA OPCIÓN | Sí, ¿con quién hablaste?…  pareja o novia(o)…………….…..….............  madre...........................................................  padre............................................................  enfermera o médico………………...…........  maestro(a)…………………………………....  amigo(a) ……………………………..……....  Con nadie …………………………………………...  **No quiero contestar ………………………………** | 1  2  3  4  5  6  7  **8** |
| 1. Hablaste con alguien sobre qué método usar después de la primera relación?   PUEDES MARCAR MAS DE UNA OPCIÓN | Sí, con quién hablaste…  pareja o novia(o)…………….…..….............  madre...........................................................  padre............................................................  enfermera o médico………………...…........  maestro(a)…………………………………....  amigo(a) ……………………………..……....  Con nadie …………………………………………...  **No quiero contestar ………………………………** | 1  2  3  4  5  6  7  **8** |
| 1. En esa primera relación sexual, ¿tú o tu pareja usaron algún método para evitar un embarazo o alguna infección de transmisión sexual? | Sí………………………………….…………….…....  No……………………………………………..……...  **No quiero contestar ………………………………** | 1 PASA A 6.24  2  **3** |
| 1. ¿Por qué no usaste algún método anticonceptivo o método para evitar alguna infección de transmisión sexual?   PASE A 6.25 | No sabíamos como adquirirlo………….…..…...…  No lo habíamos planeado…………………..……...  No teníamos dinero…………………….…..…….…  No conocíamos su existencia……………..………..  No sabíamos usarlo………………………..………..  Teníamos pena para usarlo………………..………  Mi novio(a) no quiso usarlo……………..………….  Yo no quise usarlo………………………..………....  No creíamos que fuera necesario (útil)……..……..  No pensé que nos fuéramos a embarazar……..….  Porque era una relación sexual con alguien del mismo sexo………………...…..……......………....  No lo consideramos………………..……….………..  Otro …………………………………………………..  **No quiero contestar ………………………………** | 01  02  03  04  05  06  07  08  09  10  11  12  77  **88** |
| 1. ¿Cuál método anticonceptivo usaron? | Óvulo ………………………………………….....…...  Espumas…………………………………………....…...  Pastillas………………………………………..…...…  Retirarse antes de eyacular………………………...  Condón ………………………………………..…...…  Anticonceptivos de emergencia ……………....…...  Pastillas (hormonales)………………………..….....  DIU o dispositivo……………………………....…...  Implante hormonal (también llamado  norplant o implanón)…………………..…..………...  Inyección hormonal…………………………..…......  Parche hormonal………………………….….…......  Ritmo…………………………………………..….......  Billings………………………………………….….....  Temperatura………………………………….....…...  Anticoncepción de emergencia…………………...  Ninguno………………………………….…...….......  **No quiero contestar ………………………………** | 01  02  03  04  05  06  07  08  09  10  11  12  13  14  15  16  **88** |

| **6.24b En tu relación de noviazgo actual o última relación de noviazgo, ¿Quién decide (decidía) sobre los siguientes aspectos…** | **Yo** | **Él (ella)** | **Ambos** | **No quiero contestar** |
| --- | --- | --- | --- | --- |
| a) Cuándo tener relaciones sexuales | 1 | 2 | 3 | **4** |
| b) Qué anticonceptivos usar | 1 | 2 | 3 | **4** |

**ANTECEDENTE DE EMBARAZO**

| 1. ¿Alguna vez has estado embarazada?   HOMBRE:  ¿Alguna vez has embarazado a alguien? | Sí……………………………………………….….….  Sí, embarazada actualmente………………………  No………………………………………………….…  No se……………………………………………  **No quiero contestar ………………………………** | 1 PASA A 6.28  2 PASA A 6.28  3  8  **9** |
| --- | --- | --- |
| HOMBRES PASA A 6.28  MUJERES CON INICIO DE VIDA SEXUAL (PREGUNTA 6.14 CÓDIGOS 1, 2 Y 3 ) Y QUE NO HA ESTADO EMBARAZADA (PREGUNTA 6.25 CÓDIGO 3) CONTINUE, SINO PASA 6.28 | | |
| 1. ¿Te gustaría tener hijos e hijas? | Sí………………………………….…………….…....  No……………………………………….………...…..  No sé……………………………….……………..….  **No quiero contestar ………………………………** | 1  2 PASA A 6.36  8  **9** |
| 1. ¿Cuánto tiempo te gustaría esperar para tener un hijo o hija? | \|___I___\| años  Menos de un año……………………………00  No se……………………………….…………88 | PASA A 6.36 |
| 1. ¿Cuántas veces has estado embarazada?   *(Considere embarazo actual)*  HOMBRE: ¿Cuántas veces has embarazado a alguien? | Número de embarazos \|____\|____\|  **No quiero contestar ………………………………** | **Rango 1 a 15 y 99**  **99** |
| 1. En el momento en que te/se embarazaron en la última ocasión… | querías embarazarte?............................................  querías esperar más tiempo?.................................  no querías embarazarte…......................................  **No quiero contestar ………………………………** | 1  2  3  **9** |
| 1. ¿Qué pasó con el último embarazo? | Actualmente está embarazada.…………….……..  Aborto inducido………………………………….…..  Aborto involuntario………………………………….  Nacido vivo……………………………………….….  No lo sé……….……………………………………...  **No quiero contestar ………………………………** | 1  2  3  4  **5**  **9** |
| **HOMBRES PASA PREG 6.36** | | |
| 1. En total, ¿Cuántos hijos e hijas que nacieron vivos has tenido? | Total de hijos vivos \|____\|____\|  **No quiero contestar ………………………………** | **Rango 0 a 5 y 99**  **99** |
| 1. En total, ¿Cuántos hijos e hijas que nacieron muertos has tenido? | Total de hijos muertos \|____\|____\|  **No quiero contestar ………………………………** | **Rango 0 a 5 y 99**  **99** |
| 1. En total, ¿Cuántos ABORTOS has tenido? | Total de abortos \|____\|____\|  **No quiero contestar ………………………………** | **Rango 0 a 5 y 99**  **99** |
| 1. ¿Además de los hijos que has tenido, te gustaría tener otro?   SI ACTUALMENTE ESTÁ EMBARAZADA PREGUNTE:  *¿Además del hijo que estas esperando, te gustaría tener otro?*  SI HA ESTADO EMBARAZADA PERO NO TIENE HIJOS VIVOS PREGUNTE:  *¿Te gustaría tener hijos e hijas?* | Sí………………………………….……………...1  No………………………………………….……..2  No sé……………………………….……..….….8  **No quiero contestar ………………:…………9** | PASA A 6.36 |
| 1. ¿Cuánto tiempo te gustaría esperar para tener el siguiente hijo?   SI ACTUALMENTE ESTÁ EMBARAZADA PREGUNTE:  *Después del nacimiento del hijo que estás esperando, ¿cuánto tiempo te gustaría esperar para tener el siguiente hijo?*  SI HA ESTADO EMBARAZADA PERO NO TIENE HIJOS VIVOS PREGUNTE:  *¿Cuánto tiempo te gustaría esperar para tener un hijo o hija?* | \|___I___\| años  Menos de un año……………………………00  No se……………………………….…………88  **No quiero contestar ………………………99** | **Rango 0 a 30 88 y 99** |

**Las siguientes son preguntas sobre las relaciones sexuales con penetración vaginal o anal**

| 1. Durante toda tu vida, ¿con quién has tenido relaciones sexuales? | Mujeres…………………………………………..…..  Hombres…………………………………….……….  Mujeres y Hombres…………………………..……..  **No quiero contestar ………………………………** | 1  2  3 | |
| --- | --- | --- | --- |
| 1. Durante tu vida ¿con cuántas personas distintas has tenido relaciones sexuales? | NÚMERO DE PERSONAS………….I___I | **Rango 1 a 9** | |
| 1. Durante tu vida, ¿aproximadamente cuántas veces has tenido relaciones sexuales? | NÚMERO DE VECES………….I___I___I | **Rango 1 a 50** | |
| 1. En el último año ¿has tenido relaciones sexuales? | Sí……………………………………………….…….  No……………………………………………………  **No quiero contestar ………………………………** | 1  2 PASE A 6.43  **3** | |
| 1. En los últimos tres meses, ¿has tenido relaciones sexuales? | Sí……………………………………………….…….  No……………………………………………………  **No quiero contestar ………………………………** | 1  2  **3** | |
| 1. ¿Con qué frecuencia tú y tu pareja usan (o tú y tu última pareja usaban) algún método para evitar embarazos? | Siempre………………….……………..……………  Casi siempre...……………………………….………  A veces …………………….………………..………  Nunca……………………….………………………….  **No quiero contestar ………………………………** | 1  2  3  4  **5** | |
| 1. ¿Con qué frecuencia tú y tu pareja usan (o tú y tu última pareja usaban) algún método para evitar contagiarse de alguna infección sexual? | Siempre………………….……………..……………  Casi siempre...……………………………….………  A veces …………………….………………..………  Nunca……………………….………………………….  **No quiero contestar ………………………………** | 1  2  3  4  **5** | |
| 1. En la última relación sexual, ¿Qué métodos anticonceptivos usaron tú o tu pareja?   **PUEDES SELECCIONAR MÁS DE UNA OPCIÓN** | Óvulo …………………………………...……….….…  Espumas…………………………………..…….….……  Pastillas……………………………………….….……  Retirarse antes de eyacular………….……….….….  Anticonceptivos de emergencia.……………….…..  Pastillas (hormonales)……………….……….….….  Condón…………………………………………….….  DIU o dispositivo……………………………….….….  Implante hormonal (también llamado norplant o implanón) ………………………….….….….….….…  Inyección hormonal…………………………….….…  Parche hormonal…………………………….….…..  Calendario …………………………………….….…  Ninguno………………………………….…….….…..  No quiero contestar…………………………….….... | 01  02  03  04  05  06  07 PASA A 6.45  08  09  10  11  12  13  14  88 PASA A 6.46 | |
| 1. ¿Y además utilizaron condón en tu última relación sexual? | Sí……………………………………………………..  No………………………………………………….….  No sé………………………………………….…….  No quiero contestar…………………………….... | 1  2  8 PASA A 6.46  9 | |
| 1. ¿Por qué usaron condón?   **Responde sí o no según sea el caso** | Para prevenir infección por VIH............................  Para prevenir una infección de transmisión sexual diferente al VIH.........................................  Para prevenir un embarazo no deseado..............  Porque me lo pide mi pareja aunque  desconozco el motivo...........................................  Otro (especifica)_________________________ | **Sí** | **No No quiero**  **contestar** |
|  |  | **1** | **2 3** |
|  |  | **1** | **2 3** |
|  |  | **1** | **2 3** |
|  |  | **1** | **2 3** |
|  |  |  | |
| 1. ¿Has tenido alguna experiencia en que el condón se rompa durante la relación sexual? | Sí……………………………………………………..  No………………………………………………….….  No quiero contestar…………………………….... | 1  2  9 | |
| 1. En esa última relación sexual (vaginal o anal), ¿habías tomado alcohol o usado alguna droga? | Sí……………………………………………………..  No………………………………………………….….  No se………………………………………….…….  No quiero contestar…………………………….... | 1  2  8  9 | |

Distribución de condones y uso de servicios de salud sexual y reproductiva

Las siguientes preguntas están relacionadas con el uso de servicios de salud sexual y reproductiva y el acceso a condones

| 1. ¿Alguna vez has visitado un centro de salud, clínica, hospital o doctor de cualquier tipo para recibir servicios o información sobre anticonceptivos, embarazo, aborto o enfermedades transmitidas sexualmente? | Sí……………………………………………….…….  No……………………………………………………  **No quiero contestar ………………………………** | 1  2 PASA A 6.54  **3** |
| --- | --- | --- |
| Cuando visitaste un centro de salud, clínica, hospital o al doctor, para recibir servicios o información sobre anticonceptivos, embarazo, aborto o infecciones transmitidas sexualmente   1. ¿Fuiste a una plática sobre anticonceptivos? | Sí……………………………………………….…….  No……………………………………………………  **No quiero contestar ………………………………** | 1  2  **3** |
| 1. El médico o la enfermera, ¿te habló sobre…   **PUEDES SELECCIONAR MÁS DE UNA OPCIÓN** | 1. anticonceptivos?.......................................... 2. condones?................................................... 3. anticoncepción de emergencia o pastilla del día después? ...................................................... 4. enfermedades transmitidas sexualmente?.... 5. el embarazo? .............................................. 6. No quiero contestar ………………………… | 1  2  3  4  5  6 |
| 1. ¿Pediste anticonceptivos? | Sí……………………………………………….…….  No……………………………………………………  **No quiero contestar ………………………………** | 1  2  **3** |
| 1. ¿Te sentiste lo suficientemente cómodo(a) para hacer preguntas? | Sí……………………………………………….…….  No……………………………………………………  **No quiero contestar ………………………………** | 1  2 PASA A 6.54  **3** |
| 1. ¿Te contestaron tus preguntas y te resolvieron tus dudas de manera adecuada durante la consulta? | Sí……………………………………………….…….  No……………………………………………………  **No quiero contestar ………………………………** | 1  2  **3** |
| 1. En los últimos 12 meses, ¿has recibido condones de forma gratuita? | Sí………………………………….…………………..  No………………………………….………………….  No se………………………………………………….  No quiero contestar…………………..…………….. | 1  2  8 PASA A 6.57  9 |
| 1. Aproximadamente, ¿cuántos condones te dieron gratuitamente en los últimos 12 meses? | l___I___I NUMERO DE CONDONES  No quiero contestar ……………………………… | 99 |
| 1. ¿En dónde te dieron los condones gratuitos? | Seguro Social (IMSS)...........................................  ISSSTE.................................................................  ISSSTE Estatal....................................................  Pemex..................................................................  Defensa................................................................  Marina...................................................................  Centro de Salud u Hospital de la SSA..................  IMSS Oportunidades............................................  Consultorios dependientes de farmacias.............  Médico privado.....................................................  Organizaciones No Gubernamentales................  En tu escuela.......................................................  Eventos masivos..................................................  Ferias de salud.....................................................  Establecimientos públicos....................................  Otro lugar ……………………………………..…….  No se………………………………...………..……..  No quiero contestar ……………………………… | 01  02  03  04  05  06  07  08  09  10  11  12  13  14  15  77  88  99 |

Principales fuentes de información sobre educación sexual

Las y los adolescentes aprenden sobre la pubertad de diferentes fuentes, personas o lugares.

| 1. ¿Cuál ha sido la fuente más importante de información para ti sobre la pubertad?   (por pubertad queremos decir las maneras en que los cuerpos de los niños y las niñas cambian durante la adolescencia) | Maestro/a………………………………………...….  Madre……………………………………………...…  Padre…………………………………………...……  Hermano………………………………………...…..  Hermana……………………………………...……..  Otros miembros de la familia………………...…...  Doctores……………………………………...……..  Libros/revistas………………………………...…....  Películas/videos………………………………...….  Internet………………………………………...…….  Otro…………………………………………….....…  **No quiero contestar ………………………………** | 01  02  03  04  05  06  07  08  09  10  77  **99** |
| --- | --- | --- |
| 1. ¿Cuál ha sido la fuente más importante de información para ti sobre los sistemas reproductivos y sexuales de los hombres y las mujeres?   Es decir, dónde se crean los óvulos y espermatozoides y cómo sucede el embarazo. | Maestro/a………………………………………...….  Madre……………………………………………...…  Padre…………………………………………...……  Hermano………………………………………...…..  Hermana……………………………………...……..  Otros miembros de la familia………………...…...  Doctores……………………………………...……..  Libros/revistas………………………………...…....  Películas/videos………………………………...….  Internet………………………………………...…….  Otro…………………………………………….....…  **No quiero contestar ……………………………** | 01  02  03  04  05  06  07  08  09  10  77  **99** |
| 1. ¿Cuál ha sido la fuente más importante de información para ti sobre cómo deben ser las relaciones entre las personas? Es decir, cómo los muchachos deben tratar a las muchachas, y vice versa. | Maestro/a………………………………………...….  Madre……………………………………………...…  Padre…………………………………………...……  Hermano………………………………………...…..  Hermana……………………………………...……..  Otros miembros de la familia………………...…...  Doctores……………………………………...……..  Libros/revistas………………………………...…....  Películas/videos………………………………...….  Internet………………………………………...…….  Otro…………………………………………….....…  **No quiero contestar ………………………………** | 01  02  03  04  05  06  07  08  09  10  77  **99** |

**Derechos sexuales y reproductivos**

| 1. ¿Cuál(es) son los derecho(s) que tienen los adolescentes…   PUEDES MARCAR MÁS DE UNA OPCIÓN | 1. Ser sexualmente activos solo si son mayores de edad……. 2. Recibir información sobre el uso del condón………………. 3. Recibir información sobre anticonceptivos en general……. 4. Recibir información sobre anticoncepción de emergencia.. 5. Recibir condones, solo si son hombres…………………….. 6. Recibir condones…………………………………….....…….. 7. Recibir anticonceptivos, solo si son mayores de 18 años… 8. Recibir anticonceptivos.…………………………………….... 9. Recibir anticoncepción de emergencia, solo si van acompañados de un adulto……………………………………..... 10. Recibir anticoncepción de emergencia………………….…. 11. Recibir educación sexual……………………………………... 12. Tener relaciones sexuales solo cuando lo deseen…….….. 13. Buscar una vida sexual placentera………………………….. 14. Decidir cuándo tener hijos…………………………………… 15. Decidir cuándo tener hijos, solo si viven en pareja………… 16. **No quiero contestar ………………………………** | 01  02  03  04  05  06  07  08  09  10  11  12  13  14  15  **16** |
| --- | --- | --- |

VII COMUNICACIÓN DE PADRES E HIJOS

| 7.1 En los últimos 6 meses, con qué frecuencia han hablado tú y tus padres sobre los siguientes temas… | Nunca | Pocas veces | Algunas veces | Muchas veces | **No quiero contestar** |
| --- | --- | --- | --- | --- | --- |
| 1. Sexo | 0 | 1 | 2 | 3 | **4** |
| 1. Cómo usar condones | 0 | 1 | 2 | 3 | **4** |
| 1. Protegerte de infecciones sexualmente transmitidas | 0 | 1 | 2 | 3 | **4** |
| 1. Protegerte del virus del SIDA | 0 | 1 | 2 | 3 | **4** |
| 1. Prevenir el embarazo | 0 | 1 | 2 | 3 | **4** |

VIII. ABUSO SEXUAL

En esta sección encontrarás preguntas sobre experiencias de abuso sexual que hayas sufrido ALGUNA VEZ en tu vida. Por favor selecciona tu respuesta.

| - 1. ¿Alguna vez alguien ha tocado tus genitales o te hizo cosas sexuales cuando tú no querías? | Sí……………………………………………….…….  No……………………………………………………  **No quiero contestar ………………………………** | 1  2 PASA A 9.1  **3 (pasar a 9.1)** |
| --- | --- | --- |
| - 1. Quien   *Puedes seleccionar más de una opción* | Novio(a)………………………..………...……..…....  Pareja / esposo(a) …………….………..…..……...  Madre ……………………………….……..…..…….  Madrastra………………………….……..…..…..…..  Padre ……………………………………..…..….…..  Padrastro …………………………..……..…..……..  Hermano ………………………….……..……….…..  Hermana …………………………..…..…………..  Vecino(a) …………………….……..…..………….  Tío ……………………………….....…..…..……….  Tía …………………………………..…..…………  Maestro(a) ………………………..…..…………...  Desconocido ………………………..……………  Otra…………………………………...…..…………. | 01  02  03  04  05  06  07  08  09  10  11  12  13  14 |
| - 1. La primera vez que te sucedió lo anterior, ¿cuántos años tenías? | I___I___I AÑOS  No me acuerdo …………………………….…… 98 | **Rango** (0 a edad actual del adolescente) |
| - 1. ¿Cuántos años tenía la persona que te hizo lo anterior? | I___I___I AÑOS  No se ……………………………………………..88 | **Rango 0 a 99** |

IX CREENCIAS SOBRE GÉNERO Y ACTITUDES HACIA LA DIVERSIDAD

| - 1. Las y los adolescentes tienen diferentes puntos de vista sobre las relaciones. Para cada punto de vista a continuación, marca si estás de acuerdo o en desacuerdo. | | De acuerdo | No sé/no estoy seguro(a) | En desacuerdo | **No quiero contestar** |
| --- | --- | --- | --- | --- | --- |
| 1. Creo que está bien si los/las adolescentes que no están casados sean novios, salgan juntos o tengan citas. | | 0 | 1 | 2 | **3** |
| 1. Creo que está bien si los/las adolescentes que no están casados se besen, abracen y se toquen. | | 0 | 1 | 2 | **3** |
| 1. Creo que no tiene nada de malo si los/las adolescentes que no están casados tengan relaciones sexuales si se quieren o se aman. | | 0 | 1 | 2 | **3** |
| 1. Creo que a veces un muchacho tiene que forzar a una muchacha a tener relaciones sexuales con él si la quiere. | | 0 | 1 | 2 | **3** |
| 1. Creo que un muchacho no respetará a una muchacha si ella acepta tener relaciones sexuales con él. | | 0 | 1 | 2 | **3** |
| 1. Creo que muchas muchachas que tienen relaciones sexuales antes de casarse lo lamentan después. | | 0 | 1 | 2 | **3** |
| 1. Creo que muchos muchachos que tienen relaciones sexuales antes de casarse lo lamentan después. | | 0 | 1 | 2 | **3** |
| 1. Creo que un muchacho y una muchacha deben tener relaciones sexuales antes de que decidan casarse, para ver si son compatibles. | | 0 | 1 | 2 | **3** |
| 1. Creo que a veces se justifica que un muchacho le pegue a su novia | | 0 | 1 | 2 | **3** |
| 1. Creo que está bien que los muchachos y las muchachas tengan relaciones sexuales si usan métodos para prevenir un embarazo. | | 0 | 1 | 2 | **3** |
| 1. Creo que la mayor parte de mis amigos que tienen relaciones sexuales con alguien usan condones regularmente. | | 0 | 1 | 2 | **3** |
| 1. Creo que es principalmente responsabilidad de la mujer asegurarse de que se use regularmente un anticonceptivo. | | 0 | 1 | 2 | **3** |
| 1. Creo que debes de estar enamorado/a con alguien antes de tener relaciones sexuales con él o ella. | | 0 | 1 | 2 | **3** |
| 1. Creo que los hombres necesitan sexo con más frecuencia que las mujeres. | | 0 | 1 | 2 | **3** |
| 9.2 ¿Cuántos de tus amigos y amigas han tenido relaciones sexuales? Dirías muchos, algunos, unos pocos o ninguno | Muchos………………………………………….……  Algunos……………………………………….………  Unos pocos (Unos cuantos) ……………….………  Ninguno……………………………………….………  No se…………………………………………….……  **No quiero contestar ………………………………** | | | 1  2  3  4  5  **6** |  |

**X. Sintomatología Depresiva**

A continuación hay una lista de emociones y situaciones que probablemente hayas sentido o tenido. Por favor escribe durante cuántos días en la semana pasada te sentiste así, o si te ocurrió casi diario en las últimas dos semanas

| 10.1 Durante las últimas 2 semanas, ¿qué tan seguido has tenido molestias debido a los siguientes problemas… | | Ningún  día | Varios  días | Más de la  mitad de  los días | | Casi  todos los  días | **No quiero contestar** |
| --- | --- | --- | --- | --- | --- | --- | --- |
| a) Poco interés o placer en hacer cosas | | 0 | 1 | 2 | | 3 | **4** |
| b) Te has sentido decaído(a), deprimido(a) o sin esperanzas | | 0 | 1 | 2 | | 3 | **4** |
| c) Has tenido dificultad para quedarte o permanecer dormido(a), o has dormido demasiado | | 0 | 1 | 2 | | 3 | **4** |
| d) Te has sentido cansado(a) o con poca energía | | 0 | 1 | 2 | | 3 | **4** |
| e) Sin apetito o has comido en exceso | | 0 | 1 | 2 | | 3 | **4** |
| f) Te has sentido mal contigo mismo(a) – o sentir que eres un fracaso o que has quedado mal contigo mismo(a) o con tu familia | | 0 | 1 | 2 | | 3 | **4** |
| g) Has tenido dificultad para concentrarte en ciertas actividades, tales como leer el periódico o ver la televisión | | 0 | 1 | 2 | | 3 | **4** |
| h) ¿Te has movido o hablado tan lento que otras personas podrían haberlo notado? o lo contrario – muy inquieto(a) o agitado(a) que has estado moviéndote mucho más de lo normal | | 0 | 1 | 2 | | 3 | **4** |
| i) Pensamientos de que estarías mejor muerto(a) o de lastimarte de alguna manera | | 0 | 1 | 2 | | 3 | **4** |
| SI MARCÓ CUALQUIERA DE LOS PROBLEMAS CONTUNÚE, SI NO PASA 11.1 | | | | | | |  |
| 10.2 ¿Qué tanta dificultad te han dado estos problemas para hacer tus actividades habituales, o llevarte bien con otras personas? | No ha sido difícil…………………  Un poco difícil…………………….  Muy difícil…………………………  Extremadamente difícil………….  **No quiero contestar …………………** | | | | 1  2  3  4  **5** | |  |

**XI Apego**

A continuación encontrarás una serie de frases sobre formas de comportarse contigo que tienen otros adolescentes de la escuela durante este curso. **Selecciona** lo que a ti te ha ocurrido durante este año y el anterior. A cada pregunta hay que dar sólo una respuesta

|  | Nunca | Pocas veces | Muchas veces | **No quiero contestar** |
| --- | --- | --- | --- | --- |
| 11.1. ¿Prefieres que otros chicos o chicas no te pidan cosas (por ejemplo, jugar, que le cuentes algo, etc.)? | 0 | 1 | 2 | **4** |
| 11 .2. ¿Te gusta mostrar afecto o cariño y tener amigos íntimos? | 0 | 1 | 2 | **4** |
| 11.3. ¿Es para ti muy importante no necesitar ayuda (ser independiente)? | 0 | 1 | 2 | **4** |
| 11.4. ¿Te sientes bien si otros chicos o chicas te ayudan? | 0 | 1 | 2 | **4** |
| 11.5. ¿Es para ti muy importante que hagas las cosas tu solo/a, sin ayuda? | 0 | 1 | 2 | **4** |
| 11.6. ¿Te gusta que otros chicos o chicas dependan de ti (por ejemplo, que te pidan cosas, que quieran jugar contigo)? | 0 | 1 | 2 | **4** |
| 11.7. ¿Sientes que los otros chicos o chicas no quieren ser buenos amigos/as tuyos? | 0 | 1 | 2 | **4** |
| 11.8. ¿Te cuesta mucho pedir cosas a otros chicos o chicas (como que te ayuden, que jueguen contigo, que te escuchen cuando tengas un problema, etc.)? | 0 | 1 | 2 | **4** |
| 11.9 ¿Te resulta fácil sentir afecto (o cariño) con otros chicos o chicas | 0 | 1 | 2 | **4** |
| 11.10. ¿Te preocupa dar tu afecto o confianza a otros chicos o chicas porque puedan hacerte daño? | 0 | 1 | 2 | **4** |
| 11.11. ¿Te resulta difícil confiar en otros chicos o chicas y contarles todo lo que te pasa? | 0 | 1 | 2 | **4** |
| 11.12. ¿Te sientes bien si no tienes amigas o amigos íntimos? | 0 | 1 | 2 | **4** |

**XII Escala de Función Reflexiva para Jóvenes**

Las siguientes frases tiene**n** por objeto evaluar el concepto que tienes de **tí** mismo(a). Por favor, contesta las frases con la respuesta que consideres más apropiada.

|  | Completa-mente en desacuerdo | En desacuerdo | Algo en desacuerdo | Algo de acuerdo | De acuerdo | Complete-mente de acuerdo | **No quiero contestar** |
| --- | --- | --- | --- | --- | --- | --- | --- |
| 12.1 La gente dice que los escucho | 1 | 2 | 3 | 4 | 5 | 6 | **7** |
| 12.2. Me confunde cuando las personas hablan de sus sentimientos. | 1 | 2 | 3 | 4 | 5 | 6 | **7** |
| 12.3. Creo que las personas son demasiado confusas para que me moleste en tratar de entenderlas | 1 | 2 | 3 | 4 | 5 | 6 | **7** |
| 12.4. Se me hace difícil ver los puntos de vista de otras personas | 1 | 2 | 3 | 4 | 5 | 6 | **7** |
| 12.5. Pongo atención a mis sentimientos | 1 | 2 | 3 | 4 | 5 | 6 | **7** |
| 12.6. En una discusión, mantengo en mente el punto de vista de la otra persona | 1 | 2 | 3 | 4 | 5 | 6 | **7** |
| 12.7. Comprender las razones de las acciones de otras personas me ayuda a perdonarlas | 1 | 2 | 3 | 4 | 5 | 6 | **7** |
| 12.8. Me gusta pensar sobre las razones de mis acciones | 1 | 2 | 3 | 4 | 5 | 6 | **7** |
| 12.9. Me he dado cuenta de que muchas veces las personas dan consejos a los demás que a ellas mismas le gustaría seguir | 1 | 2 | 3 | 4 | 5 | 6 | **7** |
| 12.10. Presto atención al impacto de mis acciones en los sentimientos de los demás | 1 | 2 | 3 | 4 | 5 | 6 | **7** |

XIII. ADICCIONES

En esta sección encontrarás preguntas relacionadas con el consumo de alcohol, tabaco y drogas. Por favor selecciona tu respuesta y recuerda que tus respuestas son estrictamente confidenciales.

| 13.1 ¿Has fumado por lo menos cien cigarrillos (5 cajetillas) de tabaco durante toda tu vida? | Sí………………………………………………..…….  No……………………………………………….……  Nunca he fumado……………………………………  No quiero contestar ……………………………… | 1  2  3 PASE a 13.4  **4 (pase a 13.4)** |
| --- | --- | --- |
| 13.2 ¿Cuántos cigarrillos fumas o fumabas y con qué frecuencia? | ....................... /___/___/ CUANTOS  Diario…………………………………….………..…  Semanal.…………………………………………..….  Mensual...………………………………………….….  Ocasional ……………………………………………..  Al menos una vez al año ……………………………  No se………………………………………………….. | 1  2  3  4  5  8 |
| 13.3 En los últimos 30 días, ¿has fumado tabaco? | Sí………………………………………………..…….  No……………………………………………….…… | 1  2 |

**Las próximas dos preguntas son sobre si tomas alcohol. Esto incluye tomar cerveza, pulque, vino, brandy, whisky, ron, tequila, coolers, presidencola. No incluye tomar unos sorbitos de vino para fines religiosas.**

| 13.4 En los últimos 12 meses, ¿tomaste alguna bebida que contenga alcohol? (cerveza, pulque, vino, brandy, whisky, ron, tequila, coolers, presidencola, etc.) No incluye tomar unos sorbitos de vino para fiestas religiosas | Sí………………………………………………..…….  No………………………..……………………….…  No quiero contestar ……………………………… | 1  2 PASE a 13.6  **3 (pase a 13.6)** |
| --- | --- | --- |
| 13.5 Durante los últimos 30 días, ¿cuántos días tomaste 5 o más bebidas de alcohol en una ocasión? | **....................... /___/___/ DIAS** | **Rango de 0 a 30** |
| 13.6 ¿Alguna vez has probado alguna droga, aunque sea ocasionalmente o sólo en fiestas? | Sí………………………………………………..…….  No……………………………………………….…….  No quiero contestar ………………………….. | 1  2  **3** |

**Opciones para finalizar un cuestionario incompleto Suspender entrevista 1 (que se pueda retomar en donde se quedó)**

**Terminar entrevista 2 (cierra el registro)**
