# Supplementary material for: Sexual and reproductive health outcomes are positively associated with comprehensive sexual education exposure in Mexican high-school students
Source: PLoS One. 2018 Mar 19;13(3):e0193780. doi: 10.1371/journal.pone.0193780 (PMC5858848; doi:10.1371/journal.pone.0193780)
Supplement: S2 Appendix — (DOCX) [file pone.0193780.s002.docx]

6. STUDENT’S DATA

CURRENT SCHOOL GRADE (year in school) | | |

I. GENERAL INFORMATION

THE FOLLOWING QUESTIONS REGARD YOUR FAMILY AND YOUR HOUSEHOLD, PLEASE SELECT THE CORRECT ANSWER

| 1. How old are you? | CURRENT AGE………………I___I___I | **Age range 15 to 18** |
| --- | --- | --- |
| 1. What is your gender? | Male …...…………………………………………  Female………………………………………...… | 1  2 |
| 1. What is your marital status | Single (a) …………………………………..…….…  Married (a) …………………………………....…..…  Separated (a) ……..………………………..…  Live with a partner …..……………..….  Divorced (a) …………………………………….… | 1  2  3  4  5 |

**IV. RELATIONSHIPS WITH PEERS AT SCHOLL AND ON YOUR FREE TIME**

**The following questions are related to the way people treated you emotionally in this past year.**

| - 1. During the last year (12 months), how frequently did one of the following happen?   **SKIP TO NEXT ITEM**  Never or almost never…..……..…….1  Sometimes ………………………….2  Frequently  (twice in one week) ……………..3  Very frequently  (more than twice in a week) …….4  **I don’t want to answer …………………… 5 (go to q:4.4)** | | - 1. Where? (If anything in question 4.1 happened)   Home…………….1  School……………2  Both….……………3 |
| --- | --- | --- |
| A) Did someone call you names?.................................. | [__] | [__] |
| B) Did someone made you feel bad?............................. | [__] | [__] |
| C) Did someone made you feel afraid?.......................... | [__] | [__] |
| D) Did someone physically hurt you?............................... | [__] | [__] |

| - 1. Select how true the following things are for you: | Never | Once in a while | Sometimes | Several times | **I don’t want to answer** |
| --- | --- | --- | --- | --- | --- |
| A) People call me names | 0 | 1 | 2 | 3 | **4** |
| B) People bother me or attack me | 0 | 1 | 2 | 3 | **4** |
| C) I like to bother weaklings | 0 | 1 | 2 | 3 | **4** |
| D) I like to fight with people I know I can easily defeat | 0 | 1 | 2 | 3 | **4** |
| E) People makes fun of me | 0 | 1 | 2 | 3 | **4** |
| F) People hit me or push me | 0 | 1 | 2 | 3 | **4** |
| G) I’m part of a group that makes fun of other people | 0 | 1 | 2 | 3 | **4** |
| H) I like other people to be afraid of me | 0 | 1 | 2 | 3 | **4** |

**Bullying due to sexual orientation and other reasons**

| - 1. How frequently do you hear expression such as “that’s so gay”, “you are so gay” at school? | | Very frequently……………………………….....  Frequently …………………………………..…  Sometimes …………………………………..….  On rare occasions …………………………..….  Never……………………………………………..….  **I don’t want to answer …………………………** | | | | 1  2  3  4  5  **6** | | |  |
| --- | --- | --- | --- | --- | --- | --- | --- | --- | --- |
| - 1. How frequently have you heard expressions such as “dyke” “faggot” or “lesbian” used in an insulting way at school? | | Very frequently……………………………….....  Frequently …………………………………..…  Sometimes …………………………………..….  On rare occasions …………………………..….  Never……………………………………………..….  **I don’t want to answer …………………………** | | | | 1  2  3  4  5  **6** | | |  |
| - 1. Would you say that they use such expressions at school: | | Most students ……………………  Some students ………………………..  A few students ………………………..…  **I don’t want to answer …………………** | | | | 1  2  3  **4** | | |  |
| - 1. De you feel insecure at school because of:   *Select all possible answers* | | a) Your sexual orientation?.....................................  b) Your gender (male or female)?..................  c) The color of your skin or ethnicity? …………  d) The way you express your gender (how traditionally “male” or “female” your are, look, and act)? ...............................................................  e) A disability that you have or people think you have? ……………………………………………….  f) Your religion or because people think you are of a certain religion? ………………………………….  g) I don’t feel insecure …………………………..  **I don’t want to answer …………………………** | | | | 1  2  3  4  5  6  7  **8** | | |  |
| - 1. In the last year, how often have others bothered you verbally (calling you bad names, threatening you, etc.) in your school because of: | Very frequently | | Frequently | Sometimes | On rare occasions | | Never | **I don’t want to answer** | |
| a) Your sexual orientation? | 4 | | 3 | 2 | 1 | | 0 | **5** | |
| b) Your gender (male or female) | 4 | | 3 | 2 | 1 | | 0 | **5** | |
| c) The color of your skin or ethnicity | 4 | | 3 | 2 | 1 | | 0 | **5** | |
| d) The way you express your gender (how traditionally “male” or “female” your are, look, and act) | 4 | | 3 | 2 | 1 | | 0 | **5** | |
| e) A disability that you have or people think you have | 4 | | 3 | 2 | 1 | | 0 | **5** | |
| f) Your religion or because people think you are of a certain religion | 4 | | 3 | 2 | 1 | | 0 | **5** | |
| - 1. In the last year, how often have others bothered you physically (pushed you, hit you, etc.) in your school because of: | Very frequently | | Frequently | Sometimes | On rare occasions | | Never | **I don’t want to answer** | |
| a) Your sexual orientation? | 4 | | 3 | 2 | 1 | | 0 | **5** | |
| b) Your gender (male or female) | 4 | | 3 | 2 | 1 | | 0 | **5** | |
| c) The color of your skin or ethnicity | 4 | | 3 | 2 | 1 | | 0 | **5** | |
| d) The way you express your gender (how traditionally “male” or “female” your are, look, and act) | 4 | | 3 | 2 | 1 | | 0 | **5** | |
| e) A disability that you have or people think you have | 4 | | 3 | 2 | 1 | | 0 | **5** | |
| f) Your religion or because people think you are of a certain religion | 4 | | 3 | 2 | 1 | | 0 | **5** | |

**SCHOOLS**

| **The following questions refer to your education during elementary-school, middle-school and high-school. Please select the answer that best applies.** | | | |
| --- | --- | --- | --- |
| - 1. During elementary school, middle-school/junior-high or high-school, did a teacher or counselor talk to you about any of the following?     Yes, a teacher …..….1  Yes, a counselor …...2  Yes, the principal …..3  GO TO THE NEXT SECTION  No…………………….…..4  **I don’t want to answer ……..5 (go to q.4.16)** | | |  |
|  | | |  |
| a) Puberty (how the male and female body changes during adolescence) | | I___I |  |
| b) The reproductive system (where ova and sperm are formed and how pregnancy occurs) | | I___I |  |
| c) Relationships (how boys should treat girls and vice versa) | | I___I |  |
| d) How to protect yourself to prevent HIV | | I___I |  |
| e) How to protect yoursef to prevent sexually transmited diseases | | I___I |  |
| f) How to protect yourself to prevent pregnancy | | I___I |  |
| g) How to use a condom | | I___I |  |
| h) How to talk to your partner about condom use | | I___I |  |
| i) How to avoid situations that could lead to unwanted intercourse or intercourse without protection | | I___I |  |
| j) The correct use of condoms and other contraceptive methods. | | I___I |  |
| k) Where to get condoms or other contraceptive methods | | I___I |  |
| l) How to overcome barriers to get condoms or other contraceptive methods | | I___I |  |
| m) Equity and gender equallity | | I___I |  |
| n) Sexual and reproductive health rights | | I___I |  |
| o) Physical violence | | I___I |  |
| p) Sexual violence | | I___I |  |
| q) Pleasure (That sex should be pleasant and it shouldn’t be forced; sexuality as part of life and each person; acceptance of masturbation) | | I___I |  |
| r) Implications of drug and alcohol use. | | I___I |  |
| s) Social relationships and values, recognizing healthy or coercive relationships | | I___I |  |
| t) Respect for ethnic diversity | | I___I |  |
| u) Respect for sexual orientation or sexual diversity | | I___I |  |
| v) Respect for people with HIV and AIDS | | I___I |  |
| x) Respect for people with disability | | I___I |  |
| y) Access to health-care services | | I___I |  |

V. INTER-PARTNER RELATIONSHIPS

In the following section you will find questions regarding how you perceive interpersonal relationships with a partner (boyfriend / girlfriend) or with sexual or emotional partners (whether you now have or used to have a partner or not) and about some situations that may have happened during your last or current relationship. Please select the answer that best applies.

| - 1. During the last year (12 months), how many times did your boyfriend/girlfriend or someone you were dating:   …insult you, told you that you were stupid, ridiculed you, made fun of you, prohibited you from having friends or started any false rumors about you? | I did not have a relationship /dated during the last year….………………………………………….….  0 times…………………………………….….……...  1 time…………………………………….….……...  2 or 3 times……………………………….….………  4 or 5 times………………………….….………   1. or more times……………………….…..…..…   **I don’t want to answer …………………………** | GO TO Q 5.7  1  2  3  4  5  6  7 |
| --- | --- | --- |
| - 1. During the last year (12 months), how many times did your boyfriend/girlfriend or someone you were dating:   …. Physically hurt you on purpose?  (Including: hitting you, pushing you, pushing you into something, hurting you with an object or weapon) | I did not have a relationship /dated during the last year….………………………………………….….  0 times…………………………………….….……...  1 time…………………………………….….……...  2 or 3 times……………………………….….………   1. or 5 times………………………….….………   6 or more times……………………….…..…..…  **I don’t want to answer…………………………..** | 1  2  3  4  5  6  **7** |
| - 1. During the last year (12 months), how many times did your boyfriend/girlfriend or someone you were dating:   … Forced you to perform sexuall actividies you did not want to do?  (Including: kissing, touching, physically forced you to have intercourse) | I did not have a relationship /dated during the last year….………………………………………….….  0 times…………………………………….….……...  1 time…………………………………….….……...   1. or 3 times………………………….….………   4 or 5 times………………………….….………  6 or more times……………………….…..…..…  **I don’t want to answer…………………………..** | 1  2  3  4  5  6  **7** |

| - 1. In your last or current relationship with a partner, who decides/decided the following? | **Me** | **Him / Her** | **Both** |
| --- | --- | --- | --- |
| c) When to visit friends | 1 | 2 | 3 |
| d) When to participate in school activities | 1 | 2 | 3 |
| e) What type of clothes you should wear | 1 | 2 | 3 |
| f) When to go out to the movies or somewhere else | 1 | 2 | 3 |
| g) When to go to a night club or party | 1 | 2 | 3 |
| h) When to cut class or miss school | 1 | 2 | 3 |

VI. ATTITUDES AND KNOWLEDGE ABOUT CONDOMS AND CONTRACEPTIVE METHODS.

In the following section we refer to the methods a couple could use to prevent a pregnancy

| 1. Have you ever heard of (METHOD)…   Yes ………………………..………………..1  GO TO NEXT SECTION  No……………………………………..….2  **I don’t want to answer …………………….3 (go to q 6.4)** | | 1. Do you know a place or person where adolescents can get this method (the one in 6.1)?   Yes……………….………..1  No……………………….2 | 1. Where did you hear about this method (the one in 6.1)?   School ................................. 1  Home ...................................... 2  Public or private health institution.............................. 3  Internet .................................. 4  Friends .................................. 5  Other……………………….7 |
| --- | --- | --- | --- |
| a) Oral contraceptives? | I___I | I___I | I___I |
| b) hormonal contraceptive injections? | I___I | I___I | I___I |
| c) Condoms? | I___I | I___I | I___I |
| d) Emergency contraception (morning after pill)? | I___I | I___I | I___I |

| 1. There are other methods that we did not mention in the previous questions, what other methods have you ever heard about?   SELECT **ALL** THAT APPLY | Pulling out…………………………………………....  Rhythm/periodic abstinence……………………..…  Oral contraceptives……………………………..…..  Injections…………………………………………...…  Condoms.………………………………………….....  Emergency contraception…………………….…....  IUD……………………………………………….…...  Hormonal patch……………………………….…..…  Spermicide……….…………………………..………  Female sterilization………….………………….….  Male sterilization (vasectomy).…………………....  Other………………………………………………..  **I don’t want to answer ……………………………** | 01  02  03  04  05  06  07  08  09  10  11  77  **99** |
| --- | --- | --- |
| 1. Which method do you consider best for an adolescent ¿Cuál método consideras que es más adecuado para ser utilizado por un(a) adolescente?   SELECT **ALL** THAT APPLY | Pulling out…………………………………………....  Rhythm/periodic abstinence……………………..…  Oral contraceptives……………………………..…..  Injections…………………………………………...…  Condom…………………………………………….....  Emergency contraception…………………….…....  IUD……………………………………………….…...  Hormonal patch ……………………………….…..…  Spermicide………..…………………………..………  Other……………………………………..………….….  I don’t know……………………………..……….…..  **I don’t want to answer ……………………………** | 01  02  03  04  05  06  07  08  09  77  88  **99** |
| 1. Do you think any method could harm you?   SELECT **ALL** THAT APPLY | Pulling out…………………………………………....  Rhythm/periodic abstinence……………………..…  Oral contraceptives……………………………..…..  Injections…………………………………………...…  Condom…………………………………………….....  Emergency contraception…………………….…....  IUD……………………………………………….…...  Hormonal patch ……………………………….…..…  Spermicide………..…………………………..………  Other……………………………………..………….….  I don’t know……………………………..……….…..  **I don’t want to answer ……………………………** | 01  02  03  04  05  06  07  08  09  10  77  88  **99** |
| 1. How many times can you use a male condom? | I___I times  I don’t know.....................................8 |  |
| 1. Are male condoms used to prevent pregnancy or sexually transmitted diseases? | Prevent pregnancy....................................  Prevent sexually transmitted diseases.................................................................  Both.............................................................  I don’t know...............................................................  **I don’t want to answer ………………………………** | 1  2  3  8  **9** |
| - 1. Which of the following do you think can prevent a sexually transmitted disease, HIV or AIDS?   SELECT **ALL** THAT APPLY | Pulling out? ……..………………….....  Oral contraceptive? ……..……..……..………...….  Douching? ……………………………….....…  Condoms? …………………………….…..……..….  Rhythm? …………………………..….…  Herbal or medicinal teas? …………………..……..  Having intercourse only with one’s partner? ………  Not having intercourse?…………..…….......  Other?.......................................................…….......  I don’t want to answer……………...…………..…... | 01  02  03  04  05  06  07  08  77  99 |

**Knowledge and attitudes related to condom use**

People can have different opinions related to condom use

| - 1. To what degree do you agree or disagree with the following statements: | Agree | Do not agree or disagree | Disagree | **I don’t want to answer** |
| --- | --- | --- | --- | --- |
| a) Condoms are an effective method to prevent pregnancy | 1 | 2 | 3 | **4** |
| b) A condom can only be used once | 1 | 2 | 3 | **4** |
| c) A woman can ask her boyfriend to use a condom | 1 | 2 | 3 | **4** |
| d) A man can ask his girlfriend to use a condom | 1 | 2 | 3 | **4** |
| e) Condoms are a effective way to protect yourself from HIV/AIDS | 1 | 2 | 3 | **4** |
| f) Using condoms is right for sporadic/occasional intercourse | 1 | 2 | 3 | **4** |
| g) Using condoms is right for stable, loving relationships | 1 | 2 | 3 | **4** |
| h) It would be embarrassing for someone like me to buy or get condoms | 1 | 2 | 3 | **4** |
| i) If a female adolescent asks her partner to use a condom it means she doesn’t trust him | 1 | 2 | 3 | **4** |
| j) The use of condoms decreases sexual pleasure | 1 | 2 | 3 | **4** |
| k) Condoms can fall off the penis and disappear into the woman’s body | 1 | 2 | 3 | **4** |
| l) If an unmarried couple wants to have sex before marriage, they should use condoms. | 1 | 2 | 3 | **4** |
| m) Condoms are an effective way to protect from sexually transmitted diseases | 1 | 2 | 3 | **4** |

**Self-efficacy regarding condom use**

| In the following section we present some situations to show how confident or unsure you are that you can do the following. There are no right or wrong ansuers. | | | | | | |
| --- | --- | --- | --- | --- | --- | --- |
| - 1. **I feel sure that...** | Very unsure | Unsure | Somewhat Unsure | Sure | Very Sure | **I don’t want to answer** |
| 1. I could carry a condom with me in case I needed one | 1 | 2 | 3 | 4 | 5 | **6** |
| 1. Talk about using condoms with any sexual partner | 1 | 2 | 3 | 4 | 5 | **6** |
| 1. I could talk about using a condom if I were not sure about my partner’s feelings regarding condoms. | 1 | 2 | 3 | 4 | 5 | **6** |
| 1. I could talk about about using condoms with a potential partner before we started kissing and hugging. | 1 | 2 | 3 | 4 | 5 | **6** |
| 1. I could convince a partner to use condoms when we have sex. | 1 | 2 | 3 | 4 | 5 | **6** |
| 1. I could say no to sex if my partner refused to use a condom | 1 | 2 | 3 | 4 | 5 | **6** |
| 1. I could use a condom every time my partner and I have sex | 1 | 2 | 3 | 4 | 5 | **6** |
| 1. I could use a new condom every time my partner and I have sex | 1 | 2 | 3 | 4 | 5 | **6** |
| 1. I could stop and put a condom on me or my partner. | 1 | 2 | 3 | 4 | 5 | **6** |
| **How confident are you that...** |  |  |  |  |  |  |
| 1. I or my partner could unroll a condom all the way to the base of the penis | 1 | 2 | 3 | 4 | 5 | **6** |
| 1. I or my partner could use a condom without it slipping | 1 | 2 | 3 | 4 | 5 | **6** |
| 1. Me or my partner could dispose a condom in the trash after having intercourse. | 1 | 2 | 3 | 4 | 5 | **6** |
| 1. I or my partner could hold the condom at the base of the penis while withdrawing after sex | 1 | 2 | 3 | 4 | 5 | **6** |
| 1. I could use a condom if drinking beer, whine or other liquor | 1 | 2 | 3 | 4 | 5 | **6** |

| **6.24b In your current or last relationship, who decided about the following aspects?…** | **Me** | **Him/Her** | **Both** | **I don’t want to answer** |
| --- | --- | --- | --- | --- |
| a) When to have sex | 1 | 2 | 3 | **4** |
| b) Which contraceptive method to use | 1 | 2 | 3 | **4** |

Condom distribution and sexual and reproductive health care use

The following questions are about the use of sexual and reproductive health care facilities and access to condoms.

| - 1. Have you ever visited a health clinic, hospital or doctor to receive any service or information regarding contraception, pregnancy, abortion or sexually transmitted diseases? | Yes………………………………………….…….  No……………………………………………………  **I don’t want to answer ……………………………** | 1  2 GO TO Q.6.54  **3** |
| --- | --- | --- |

**Reproductive and sexual rights**

| - 1. Which of the followings are the rights of adolescents?   SELECT **ALL** THAT APPLY | 1. Being sexually active only if they are adults ……. 2. Receiving information about condom use ……………. 3. Receiving information about contraception in general…. 4. Receiving information about emergency contraception.. 5. Receiving condoms only if they are male ………………… 6. Receiving condoms…………………………………….....… 7. Receiving contraception only if they are over 18 years…. 8. Receiving contraception …………………………………….. 9. Receiving emergency contraception only if they are accompanied by and adult.……………………………………..... 10. Receiving emergency contraception………………….…. 11. Receiving sexual education…………………………………... 12. To have sex only when they want to …….….. 13. To seek a pleasurable sexual life ………………………….. 14. To decide when to have children…………………………… 15. To decide when to have children only if they live with their partner ……… 16. **I don’t want to answer ………………………………** | 01  02  03  04  05  06  07  08  09  10  11  12  13  14  15  **16** |
| --- | --- | --- |

VIII. SEXUAL ABUSE

In the following section you will find some questions regarding any experience with sexual abuse you have ever had. Please select the answer that applies

| - 1. Has anyone ever touched your genitals or done something sexual to you when you didn’t want them to? | Yes…………………………………………….…….  No……………………………………………………  **I don’t want to answer …………………………** | 1  2 GO TO Q 9.1  **3 (GO TO Q 9.1)** |
| --- | --- | --- |

IX BELIEFS ABOUT GENDER AND ATTITUDDES TOWARDS DIVERSITY

| - 1. Adolescents have different opinions about relationships. Select if you agree or disagree with each one of the following statements. | Agree | I’m not sure | Disagree | **I don’t want to answer** |
| --- | --- | --- | --- | --- |
| 1. I think it is ok for unmarried adolescents to be a couple, date, or hang out. | 0 | 1 | 2 | **3** |
| 1. I think it is ok for unmarried adolescents to kiss, hug or touch. | 0 | 1 | 2 | **3** |
| 1. I believe there is nothing wrong with unmarried adolescents having sex if they love or care about each other. | 0 | 1 | 2 | **3** |
| 1. I believe that sometimes a boy has to force a girl to have sex with him if she doesn’t want to. | 0 | 1 | 2 | **3** |
| 1. I believe that a boy will not respect a girl if she agrees to have sex with him. | 0 | 1 | 2 | **3** |
| 1. I believe that girls who have sex before marriage will regret it. | 0 | 1 | 2 | **3** |
| 1. I believe that boys who have sex before marriage will regret it. | 0 | 1 | 2 | **3** |
| 1. I believe that a boy and a girl should have sex before getting married to see if they are compatible. | 0 | 1 | 2 | **3** |
| 1. I believe that sometimes is ok for a boy to hit his girlfriend. | 0 | 1 | 2 | **3** |
| 1. I believe it is ok for boys and girls to have sex if they use contraceptive methods to prevent pregnancy. | 0 | 1 | 2 | **3** |
| 1. I believe that most of my friends who have sex use condoms regularly | 0 | 1 | 2 | **3** |
| 1. I believe that it is the girl’s responsibility to make sure to use contraception regularly. | 0 | 1 | 2 | **3** |
| 1. I believe you must be in love with someone before having sex. | 0 | 1 | 2 | **3** |
| 1. I believe men need sex more frequently than women. | 0 | 1 | 2 | **3** |
